# Bioinformatic Analysis of the *Campylobacter jejuni* Type VI Secretion System and Effector Prediction

**DOI:** 10.3389/fmicb.2021.694824

**Published:** 2021-06-29

**Authors:** Luca Robinson, Janie Liaw, Zahra Omole, Dong Xia, Arnoud H. M. van Vliet, Nicolae Corcionivoschi, Abderrahman Hachani, Ozan Gundogdu

**Affiliations:** ^1^Faculty of Infectious and Tropical Diseases, London School of Hygiene & Tropical Medicine, London, United Kingdom; ^2^Comparative Biomedical Sciences, Royal Veterinary College, London, United Kingdom; ^3^School of Veterinary Medicine, Faculty of Health and Medical Sciences, University of Surrey, Guildford, United Kingdom; ^4^Bacteriology Branch, Veterinary Sciences Division, Agri-Food and Biosciences Institute, Belfast, United Kingdom; ^5^Bioengineering of Animal Science Resources, Banat University of Agricultural Sciences and Veterinary Medicine – King Michael the I of Romania, Timisoara, Romania; ^6^The Peter Doherty Institute for Infection and Immunity, Department of Microbiology and Immunology, University of Melbourne, Melbourne, VIC, Australia

**Keywords:** *Campylobacter jejuni*, Type VI Secretion System, T6SS effectors, T6SS immunity proteins, toxin-antitoxin, pathogenicity island

## Abstract

The Type VI Secretion System (T6SS) has important roles relating to bacterial antagonism, subversion of host cells, and niche colonisation. *Campylobacter jejuni* is one of the leading bacterial causes of human gastroenteritis worldwide and is a commensal coloniser of birds. Although recently discovered, the T6SS biological functions and identities of its effectors are still poorly defined in *C. jejuni*. Here, we perform a comprehensive bioinformatic analysis of the *C. jejuni* T6SS by investigating the prevalence and genetic architecture of the T6SS in 513 publicly available genomes using *C. jejuni* 488 strain as reference. A unique and conserved T6SS cluster associated with the *Campylobacter jejuni* Integrated Element 3 (CJIE3) was identified in the genomes of 117 strains. Analyses of the T6SS-positive 488 strain against the T6SS-negative *C. jejuni* RM1221 strain and the T6SS-positive plasmid pCJDM202 carried by *C. jejuni* WP2-202 strain defined the “T6SS-containing CJIE3” as a pathogenicity island, thus renamed as *Campylobacter jejuni* Pathogenicity Island-1 (CJPI-1). Analysis of CJPI-1 revealed two canonical VgrG homologues, CJ488_0978 and CJ488_0998, harbouring distinct C-termini in a genetically variable region downstream of the T6SS operon. CJPI-1 was also found to carry a putative DinJ-YafQ Type II toxin-antitoxin (TA) module, conserved across pCJDM202 and the genomic island CJIE3, as well as several open reading frames functionally predicted to encode for nucleases, lipases, and peptidoglycan hydrolases. This comprehensive *in silico* study provides a framework for experimental characterisation of T6SS-related effectors and TA modules in *C. jejuni*.

## Introduction

Bacterial secretion systems, classified from Type I to X according to their genetic and structural organisation and composition, are protein transport machineries enabling niche colonisation, interaction with host cells, and bacterial antagonism (Costa et al., 2015; Palmer et al., 2021). Genes encoding for the Type VI Secretion System (T6SS) are present in more than 25% of *Proteobacteria* (Bingle et al., 2008; Barret et al., 2013). The injection of a panel of T6SS effectors into competing bacteria promotes the fitness of T6SS-positive strains in polymicrobial environments, including the gut ecosystem (Coulthurst, 2019; Wood et al., 2020). However, the T6SS is not restricted to bacterial antagonism and can mediate host-pathogen interactions. Some T6SS effectors bear anti-eukaryotic activities that subvert the host cell cytoskeleton, evade host defences by countering reactive oxygen species (ROS), and modulate the host inflammatory response (Hachani et al., 2016; Chen et al., 2019).

**FIGURE 1:**
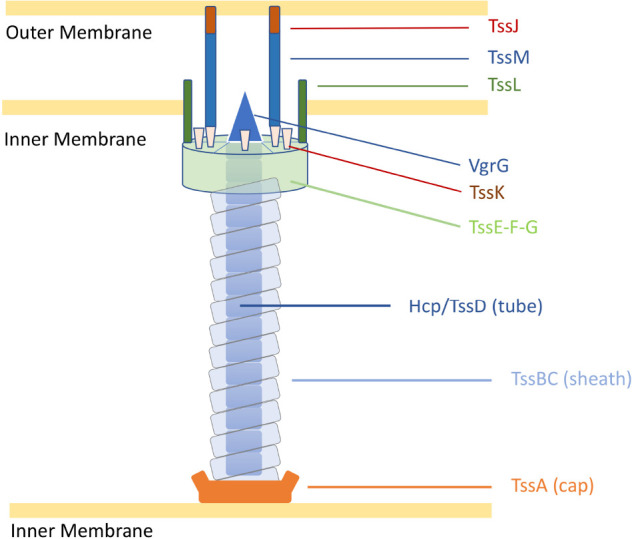
Representation of the *C. jejuni* T6SS in extended state [adapted from Cherrak et al. (2019)]. Proteins are labelled according to the nomenclature used for *C. jejuni* T6SS components. Tss, Type six secretion.

Despite the multiple roles of T6SSs in complex ecosystems, the genes encoding the T6SS core components are highly conserved into genomic clusters (Coulthurst, 2019). The structure of the T6SS shares features with the bacteriophage T4 contractile apparatus, with structural homology to the phage tail tube and spike proteins (Ho et al., 2014). A fully assembled T6SS apparatus requires a minimal set of 13 core components (Zoued et al., 2014). The machinery is characterised by a puncturing spike (VgrG) that structurally resembles the bacteriophage T4 gp27/gp5 proteins (typically sharpened by a Proline-Alanine-Alanine-Arginine (PAAR) protein), a contractile sheath (formed by the complex TssB and TssC) encasing a needle-like tube (Hcp/TssD) and capped by a core component (TssA) in the cytoplasm. A scaffold formed by a membrane-associated complex (TssJLM) and a cytoplasmic baseplate (TssEFGK) complete the system (Figure 1; Leiman et al., 2009; Zoued et al., 2014; Cianfanelli et al., 2016). Upon contraction, the TssBC sheath propels the VgrG-PAAR complex and associated effectors into target cells or the external milieu (Coulthurst, 2019). The contracted sheath can be depolymerised by the ATPase ClpV/TssH and released TssB and TssC subunits are recycled for assembly (Kapitein et al., 2013; Zoued et al., 2014).

Type VI Secretion System effectors exhibit a wide range of functions, mostly anti-bacterial, with some displaying transkingdom activities and few solely targetting eukaryotes (Alteri and Mobley, 2016). To date, T6SS effectors have been shown to harbour functions such as nucleases (Ma et al., 2014; Jana et al., 2019), lipases (Russell et al., 2013; Jiang et al., 2016), peptidoglycan hydrolases (Russell et al., 2011; Whitney et al., 2013), pore-forming activities (English et al., 2012; Fridman et al., 2020), actin cross-linking (Pukatzki et al., 2006), and anti-fungal activities (Trunk et al., 2018, 2019). Translocation of “cargo” effectors is mediated through their non-covalent interaction with Hcp, VgrG, or PAAR proteins, whilst “specialised” effectors consist of catalytic domains covalently fused to Hcp, VgrG, or PAAR proteins (Coulthurst, 2019). Anti-bacterial effector genes are often associated with cognate genes encoding for immunity proteins, producing effector-immunity pairs (Coulthurst, 2019). Immunity proteins are typically located in the cellular compartments targetted by their cognate effectors to prevent self-toxicity (Alcoforado Diniz et al., 2015).

*Campylobacter jejuni* is a gram-negative microaerophilic bacterium and one of the leading causes of human foodborne gastroenteritis worldwide (Burnham and Hendrixson, 2018). *C. jejuni* is abundant in the chicken gut, making handling and consumption of contaminated poultry products the primary foodborne transmission route to humans (Ijaz et al., 2018; McKenna et al., 2020). Although considered a commensal of the avian gut, *C. jejuni* can be an opportunistic pathogen in birds, depending on the genetics of host and bacterial strain (Humphrey et al., 2014; Wigley, 2015). *C. jejuni* infection in humans can cause bloody diarrhoea, fever, and abdominal pains. In low-resource regions, *C. jejuni* infections are common in young children and correlate with stunted growth and life-long physical and cognitive deficiencies (Amour et al., 2016). In high-resource regions, it is estimated that 1 in every 100 individuals develop a *C. jejuni*-related illness each year (Tam et al., 2012). It is still unclear how avian species can tolerate a high presence of *C. jejuni* without developing overt disease, yet a relatively low infectious dose leads to disease in humans (Gundogdu and Wren, 2020).

In *C. jejuni*, the T6SS biological functions have been associated with host colonisation, cell adhesion and invasion, survival in bile salts, contact-dependent lysis of erythrocytes, and contributing to oxidative stress (Lertpiriyapong et al., 2012; Bleumink-Pluym et al., 2013; Liaw et al., 2019). So far, Hcp has been suggested as a T6SS effector contributing to *C. jejuni* host cell adhesion and invasion (Lertpiriyapong et al., 2012; Bleumink-Pluym et al., 2013; Noreen et al., 2018); however, no evidence currently supports the direct contribution to these phenotypes by Hcp in isolation. Whilst few studies have indicated the prevalence of T6SS in *C. jejuni* (Harrison et al., 2014; Corcionivoschi et al., 2015; Ugarte-Ruiz et al., 2015), a larger comprehensive bioinformatic analysis of T6SS within *C. jejuni* and the identification of associated effectors are still needed. In this study, we investigated the prevalence and genomic organisation of the T6SS in 513 publicly available *C. jejuni* genomes through screening of the major T6SS components, previously characterised T6SS effectors, and the integrative element *Campylobacter jejuni* Integrated Element 3 (CJIE3). Using *C. jejuni* 488 strain as reference we discovered a novel pathogenicity island (PAI) variant of CJIE3, reclassifying the “T6SS-containing CJIE3” as *Campylobacter jejuni* Pathogenicity Island-1 (CJPI-1). We also observed that two distinct VgrG proteins are present in the PAI of *C. jejuni* 488 strain, with a number of other *C. jejuni* strains also possessing both VgrG proteins. Using bioinformatic analysis, we identified a number of putative T6SS effectors and predicted toxin-antitoxin (TA) modules carried by the CJPI-1 PAI.

## Materials and Methods

### Genome Sequencing, Assembly and Annotation of *C. jejuni* 488 Strain

Genome sequencing was performed as previously described by Ugarte-Ruiz et al. (2015) and adapted for this study. Briefly, paired-end Fastq data was generated on an Illumina MiSeq and assessed using FastQC (Andrews, 2010). Quality control of the sequencing reads was conducted using Trimmomatic (v0.39) (“leading” and “trailing” setting of 5, a “slidingwindow” setting of 4:20 and a “minlength” of 36 nucleotides) (Bolger et al., 2014). Assembly was performed with VelvetOptimiser (v2.2.6) using n50 optimisation and “kmer” length of 37 nucleotides (Zerbino and Birney, 2008; Gladman and Seemann, 2012). Contigs were ordered against T6SS-positive *C. jejuni* M129 strain (accession no. CP007749) (Konkel et al., 1992) using ABACAS (v1.3.1) (Assefa et al., 2009). Annotation of the genome was performed with prokka (v.1.14.6) (Seemann, 2014) using *C. jejuni* NCTC11168 strain (AL111168) (Parkhill et al., 2000). The genome was visualised and manually edited using Artemis and Artemis Comparison Tool (ACT) software (Carver et al., 2005, 2012).

### *In silico* Identification of T6SS-Containing *C. jejuni* Genomes

Nucleotide and amino acid sequences of *C. jejuni* genomes were collected from the NCBI RefSeq genome database release 99 (May 2020) at assembly level “scaffold” or higher (NCBI, 1982b). Reference genomes 108 (JX436460) (Bleumink-Pluym et al., 2013), 43431 (genome sequence from Liaw et al., 2019), and the newly assembled 488 strain were also included into the genome dataset and a local nucleotide and protein database was constructed. Our local database was then filtered to remove genomes that possessed any of the following traits: a total genome size greater than 2 Mb, no assembly protein sequence data available to download from the RefSeq database, a total assembly possessing more than 200 contigs, or the genome possessed an Average Nucleotide Identity (ANI) of less than 95%. ANI was calculated using FastANI with a fragment length of 1000 bp against the reference *C. jejuni* NCTC11168 (AL111168) strain (Jain et al., 2018). A total of 41 genomes were removed from the dataset. Metadata, including host and sample location, was collected from the NCBI BioSample database (NCBI, 1982a). Genome visualisation was performed in Artemis (Carver et al., 2012).

BLASTP (Altschul et al., 1990) was employed to identify the 13 T6SS components amongst the *C. jejuni* genomes, using default parameters. The amino acid sequences of the 13 T6SS loci from reference strain 108 (JX36460), which carries a functional T6SS, was aligned against a local protein dataset created for the *C. jejuni* genomes (Bleumink-Pluym et al., 2013). A similarity percentage was calculated by dividing the bit-score value for each amino acid alignment by two times the specific lengths of the individual query amino acid sequence (Fridman et al., 2020). Protein presence was regarded positive when a minimum of 50% similarity was observed. *C. jejuni* genomes possessing at least 11 out of the 13 T6SS loci were considered to contain a T6SS (T6SS-positive), and those that possessed fewer than 11 were considered lacking a T6SS (T6SS-negative).

### Identification of PAAR-Containing Proteins and Characterised T6SS Effectors

BLASTP (Altschul et al., 1990) was employed to identify the presence of PAAR-motif containing proteins and known T6SS effectors in the local *C. jejuni* genome database. Representative amino acid sequences from the NCBI-CDD were downloaded for the protein subclasses PAAR1, PAAR2, PAAR3, PAAR4, PAAR5, PAAR-Rhs, PAAR-CT1, and PAAR-CT2 (Marchler-Bauer et al., 2017; Supplementary Data 1). Amino acid sequences characterised as T6SS “cargo” effectors were downloaded from the NCBI Protein database (NCBI, 1982c; Supplementary Table 1). Proteins designated as “cargo” are independent effectors that do not exist as toxin domain-containing extensions of the major T6SS components Hcp, VgrG or PAAR. The amino acid sequences were aligned against a local protein dataset created for the *C. jejuni* genomes. A minimum threshold expected value of 1e-10 was implemented in the search where a value below this threshold was deemed a positive hit.

### *In silico* Identification of CJIE3-Containing Genomes

To identify the presence of CJIE3 in the *C. jejuni* genome dataset, the gene *cje1094* (integrase) from reference strain RM1221 (CP000025) [denoted as a suitable candidate for PCR-identification of CJIE3 by Parker et al. (2006)], and genes *cje1105* and *cje1153*, were used in an *in silico* identification method. BLASTN (Altschul et al., 1990) was employed to align the nucleotide sequence of the genes against the local nucleotide dataset created for the *C. jejuni* genomes. A similarity percentage was calculated according to Fridman et al. (2020). To be regarded as positive for CJIE3, a minimum similarity of 50% was required to two of the three genes: *cje1094*, *cje1105*, and/or *cje1153*. To be regarded as possessing a T6SS-harbouing plasmid, a minimum similarity of 50% was required to only gene *cje1094* and the presence of at least 11 T6SS loci (T6SS-positive). *C. jejuni* 108 strain was excluded from this analysis as it does not possess a whole genome sequence, thus reducing this analysis to 512 genomes.

### Comparative Analysis and Alignment of *C. jejuni* Genomes and Plasmids

Artemis Comparison Tool and Clinker (Carver et al., 2005; Gilchrist and Chooi, 2021) were used to comparatively align the genome of the re-sequenced and assembled T6SS-positive *C. jejuni* 488 strain against strain RM1221 (CP000025) and annotated plasmid pCJDM202 (CP014743) (Fouts et al., 2005; Marasini and Fakhr, 2016). Amino acid identity was calculated with BLAST Global Alignment–Protein (Needleman-Wunsch Global Align), using default parameters (Altschul et al., 1990).

### Functional Analysis of Predicted Proteins in CJPI-1

Webtools NCBI CDD-BLAST (Marchler-Bauer et al., 2017), SMART (Letunic and Bork, 2018), HmmScan (Potter et al., 2018), Pfam (El-Gebali et al., 2019), SCANPROSITE (de Castro et al., 2006), CDART (Geer et al., 2002), SUPERFAMILY (Gough et al., 2001), InterPro (Mitchell et al., 2019), and MOTIF (GenomeNet, 2015) were used to identify protein domains and characteristic motifs in the CJPI-1 predicted proteins. Signal peptides, transmembrane helices, and subcellular localisation were predicted using Psortb v3.0 (Yu et al., 2010), CELLO v2.5 (Yu et al., 2006), SignalP-5.0 (Armenteros et al., 2019), TMPred (Hofmann and Stoffel, 1993), and TMHMM (Sonnhammer et al., 1998) to assist in protein function prediction. Default parameters were used throughout, with an expected value (*E*-value) of 0.01 determined as a cut-off and organism group defined as Gram-negative where required. Protein functions were inferred following congruent predictions from at least 5 out of the 9 used webtools (Supplementary Tables 2, 3). Structural homology modelling was performed using the Phyre2 and I-Tasser servers (Zhang, 2008; Kelley et al., 2015).

### Characterisation of *vgrG* Genes in *C. jejuni* 488 Strain

To assess sequence identity, amino acid sequences for proteins encoded by *vgrG1* and *vgrG2* in *C. jejuni* 488 strain were analysed by BLASTP (Altschul et al., 1990) against the NCBI reference protein database, excluding *C. jejuni* to prevent self-hits. Multiple sequence alignment of *vgrG1* and *vgrG2* was conducted using Clustal Omega (Madeira et al., 2019).

BLASTN (Altschul et al., 1990) was employed to identify *vgrG* genes amongst the *C. jejuni* genomes. The nucleotide sequence of the *vgrG* gene from the T6SS-positive 108 strain (JX436460) was aligned against a local nucleotide dataset created for the T6SS-positive *C. jejuni* genomes of assembly level “complete” or higher (Bleumink-Pluym et al., 2013). A cut-off similarity percentage was calculated according to Fridman et al. (2020). To be regarded as positive for a *vgrG* gene, a minimum similarity of 50% was required. Full length VgrG protein sequences were then obtained from T6SS-positive assembly level genomes “complete” or higher and aligned using MUSCLE (Edgar, 2004) with default parameters. Genomes CJ017CC464, CJ018CCUA, and ZS007 were removed from the analysis due to disrupted open reading frames (ORFs). A phylogenetic tree was constructed from the alignment file using the Maximum-Likelihood method, with JTT modelling, partial deletion (95%), and bootstrapping (*n* = 500) parameters, conducted in the Molecular Evolutionary Genetics Analysis X (MEGAX) v. 10.1.8 software package (Kumar et al., 2018). The analysis contained 36 amino acid sequences.

### Identification of Catalytic Residues in Putative T6SS Effectors

The amino acid sequences of the query proteins were searched against the NCBI-CDD (Marchler-Bauer et al., 2017), and the subsequent output alignments corresponding to identified domains were extracted and annotated to identify the conserved catalytic residues described (Zhang et al., 2012; Sun et al., 2015; Tak et al., 2019).

### Prevalence of CJPI-1 Functionally Predicted Proteins Within the *C. jejuni* Protein Database

BLASTP (Altschul et al., 1990) was employed to identify the presence and genomic context of the CJPI-1 functionally predicted proteins amongst the *C. jejuni* genome database. The amino acid sequences were aligned against the local protein dataset created for the *C. jejuni* genomes. A similarity percentage was calculated according to Fridman et al. (2020). Protein presence regarded as positive required a minimum similarity of 50%. *C. jejuni* 108 strains was excluded from this analysis as it does not possess a whole genome sequence, therefore this analysis involved 512 genomes.

## Results and Discussion

### Prevalence of the T6SS in *C. jejuni*

We have determined the prevalence of the T6SS in publicly available *C. jejuni* genomes (Supplementary Table 4) by compiling a local dataset of nucleotide and amino acid sequences from isolates with an assembly level “scaffold” or higher from the NCBI RefSeq genome database. This was further populated with *C. jejuni* 108 and 43431 reference strains and the newly assembled 488 strain, creating a total of 513 genomes. The prevalence study of 13 T6SS core components (TssA-TssM) against our local *C. jejuni* database classified 136 of the 513 (26.51%) *C. jejuni* genomes as T6SS-positive and 377 of the 513 (73.49%) as T6SS-negative (Supplementary Tables 5, 6). Interestingly, two T6SS-negative *C. jejuni* strains, 255 and 10186, were found to possess 10 out of the 13 T6SS genes, with the genes *tagH*, *tssG*, and *vgrG* missing. Furthermore, the genome of *C. jejuni* OXC6589 strain was identified as the only one without a T6SS complete cluster to present the gene *hcp*. Our analysis of *C. jejuni* strains identified a single copy of the T6SS operon, with a conserved set of T6SS core genes sharing synteny with closely related species (Table 1). To date, all T6SS core structural components have been identified in *C. jejuni* with the exception of TssH (ClpV), the ATPase responsible for disassembly of the contracted sheath components, which is absent from all sequenced *C. jejuni* T6SS operons. This raises the possibility of an alternative mode of sheath disassembly, or the existence of a ClpV-like ATPase encoded distally from the T6SS cluster (Liaw et al., 2019).

**TABLE 1:** *C. jejuni* and *C. coli* strains studied to date with a T6SS.

**Strain**	**Source**	**Country**	**References**
*C. jejuni* 488	Human	Brazil	Liaw et al., 2019
*C. jejuni* 43431	Human	Canada	Penner et al., 1983
*C. jejuni* RC039	Chicken	United Kingdom	Corcionivoschi et al., 2015
*C. jejuni* 108	Human	United Kingdom	Bleumink-Pluym et al., 2013
*C. jejuni* 414	Bank vole	United Kingdom	Lertpiriyapong et al., 2012
*C. coli* RM2228	Chicken	United States	Bleumink-Pluym et al., 2013

### Absence of Characterised T6SS Effectors in *C. jejuni* Genomes

Hitherto, no T6SS-associated effectors have been identified and/or characterised in T6SS-positive *C. jejuni* (Lertpiriyapong et al., 2012; Bleumink-Pluym et al., 2013; Liaw et al., 2019). Using 40 characterised ‘cargo’ effectors from a range of bacteria including *Pseudomonas aeruginosa*, *Serratia marcescens*, *Yersinia pseudotuberculosis*, and *Burkholderia thailandensis* (Supplementary Table 1), we performed BLASTP-homology searches for the presence of such effectors within *C. jejuni* strains. Searches returned no positive matches leading us to conclude that T6SS-containing *C. jejuni* may possess a subset of unique “cargo” effectors.

### T6SS-Containing *Campylobacter jejuni* Integrated Element 3 Represents a Novel Pathogenicity Island Variant

The initial study of the T6SS in *C. jejuni* revealed its integration within the earlier acquired CJIE3; a genomic island displaying a mosaic gene arrangement and present in a number of *C. jejuni* strains, including RM1221 (a T6SS-negative *C. jejuni* strain) (Fouts et al., 2005; Bleumink-Pluym et al., 2013). Distribution analyses of CJIE3 in *C. jejuni* from human and avian isolates report varying prevalence of this integrated element, with only 10% of CJIE3 harbouring a T6SS (Bleumink-Pluym et al., 2013; Kovanen et al., 2019). In this study, we screened the CJIE3 integrase, *cje1094*, and genes *cje1105* and *cje1153*, using an *in silico* identification method against a local *C. jejuni* database as proxies for CJIE3 identification (Supplementary Tables 8–10). Integrase *cje1094* possesses strong homology to *A0W69_09480* harboured on the T6SS-positive megaplasmid pCJDM202 (Table 2), therefore, we included two further proxies to distinguish between T6SS-containing CJIE3 and T6SS-harbouring plasmids. We observed that 146 of the 512 (28.51%) genomes possessed the CJIE3, of which 117 (80.14%) were T6SS-positive (Supplementary Table 4). Therefore, 117 of the 135 (86.67%) T6SS-positive genomes were identified to possess the CJIE3 and 15 (2.93%) were found harbouring T6SS-positive plasmids (Table 3). Integration of the *C. jejuni* T6SS was described as occurring between homologues of the genes *cje1139* and *cje1141/cje1142* from the CJIE3 in RM1221 (Bleumink-Pluym et al., 2013). The genes *cje1141* and *cje1142* share homology to the major T6SS component *tssI/vgrG* and possess *rhs* (rearrangement hotspots) signatures, suggested to have mediated the integration of the T6SS into CJIE3 (Hill, 1999; Jackson et al., 2009; Bleumink-Pluym et al., 2013). Our data supports the findings that the T6SS has been integrated into the CJIE3, as a significant proportion of the T6SS-positive strains in this study also possess the CJIE3. Furthermore, we identified a number of CJIE3-positive genomes that do not possess a complete T6SS cluster, further supporting that integration of the T6SS has occurred subsequently to the acquisition of the integrated element (Supplementary Table 4; Bleumink-Pluym et al., 2013).

**TABLE 2:** Predicted proteins in CJPI-1 of T6SS-positive *C. jejuni* 488 strain with the respective amino acid length and inferred function. Protein locus tags of homologous proteins found in the CJIE3 of RM1221 and pCJDM202 of WP2-202 are shown with the respective amino acid (aa) identity (%).

**Locus (488)**	**Length (AA)**	**Putative function**	**Locus (RM1221)**	**AA identity (%) with RM1221**	**Locus (pCJDM202)**	**AA identity (%) with pCJDM202**
CJ488_0928	35		CJE1092	97	−	−
CJ488_0929	116		CJE1093	100	A0W69_09485	63
CJ488_0930	312	*Integrase/Recombinase*	CJE1094	70	A0W69_09480	97
			CJE1095	26		
CJ488_0931	76		CJE1096	100	A0W69_09475	100
CJ488_0932	64	*WGR domain-like containing protein*	−	−	A0W69_09470	81
CJ488_0933	38		CJE1097	97	−	−
CJ488_0934	47		CJE1098	83	−	−
CJ488_0935	69	*Fic domain-containing protein*	CJE1100	68	−	−
CJ488_0936	46		CJE1101	57	−	−
CJ488_0937	75	*DinJ*	CJE1102	97	A0W69_09400	99
CJ488_0938	93	*YafQ endoribonuclease toxin*	CJE1103	92	A0W69_09395	99
CJ488_0939	40		−	−	−	−
CJ488_0940	52		CJE1105	28	−	−
CJ488_0941	99		CJE1105	51	−	−
CJ488_0942	58		CJE1106	43	−	−
CJ488_0943	70		CJE1106	52	−	−
CJ488_0944	572	*Conjugative transfer TraG-like protein*	CJE1107	92	A0W69_09385	58
CJ488_0945	267		CJE1109	97	A0W69_09380	98
CJ488_0946	433		CJE1110	67	A0W69_09375	67
			CJE1111	89	A0W69_09045	89
CJ488_0947	1140		CJE1112	20	A0W69_09050	98
			CJE1113	67		
CJ488_0948	359		CJE1114	74	A0W69_09055	94
					A0W69_09360	67
CJ488_0949	259		−	−	−	−
CJ488_0950	167		−	−	−	−
CJ488_0951	46		−	−	−	−
CJ488_0952	167		−	−	−	−
CJ488_0953	169		−	−	−	−
CJ488_0954	192		−	−	−	−
CJ488_0955	506		−	−	−	−
CJ488_0956	90		−	−	−	−
CJ488_0957	507	*Lipase (class 3) domain-containing protein*	CJE1115	64	A0W69_09355	62
CJ488_0958	130		–	–	–	–
CJ488_0959	178		–	–	–	–
CJ488_0960	41		–	–	–	–
CJ488_0961	225	*Lipase (class 3) domain-containing protein*	–	–	–	–
CJ488_0962	195	*Phage lysozyme-like containing protein*	–	–	–	–
CJ488_0963	119		–	–	–	–
CJ488_0964	1310		CJE1137	98	A0W69_09370	94
CJ488_0965	422		CJE1138	93	A0W69_09375 A0W69_09045	74 54
CJ488_0966	299	*TagH*	CJE1139	100	A0W69_09040	100
CJ488_0967	1175	*TssM*	–	–	A0W69_09035	100
CJ488_0968	171	*Hcp*	–	–	A0W69_09030	99
CJ488_0969	257	*TssL*	–	–	A0W69_09025	100
CJ488_0970	465	*TssK*	–	–	A0W69_09020	100
CJ488_0971	148	*TssJ*	–	–	A0W69_09015	99
CJ488_0972	415	*TssA*	–	–	A0W69_09010	99
CJ488_0973	161	*TssB*	–	–	A0W69_09005	100
CJ488_0974	484	*TssC*	–	–	A0W69_09000	100
CJ488_0975	130	*TssE*	–	–	A0W69_08995	99
CJ488_0976	573	*TssF*	–	–	A0W69_08990	100
CJ488_0977	302	*TssG*	–	–	A0W69_08985	99
CJ488_0978	883	*VgrG1*	CJE1141	53	A0W69_08980	76
CJ488_0979	317	*Ankyrin domain containing protein*	–	–	–	–
CJ488_0980	438	*Tox-REase-7 domain containing protein*	–	–	–	–
CJ488_0981	189		–	–	–	–
CJ488_0982	564	*Tox-REase-7 domain containing protein*	–	–	–	–
CJ488_0983	241	*Ankyrin-like protein*	–	–	–	–
CJ488_0984	53		–	–	–	–
CJ488_0985	154		–	–	–	–
CJ488_0986	416		–	–	–	–
CJ488_0987	116		–	–	–	–
CJ488_0988	121	*TNT domain-containing protein*	–	–	–	–
CJ488_0989	184		–	–	–	–
CJ488_0990	178		–	–	A0W69_08930	49
CJ488_0991	90		–	–	–	–
CJ488_0992	128		–	–	–	–
CJ488_0993	185		–	–	–	–
CJ488_0994	123	*AHH-nuclease domain-containing protein*	–	–	A0W69_08930	41
CJ488_0995	188		–	–	A0W69_08925	86
CJ488_0996	116	*DUF4299 family protein*	–	–	A0W69_08920	100
CJ488_0997	210		–	–	A0W69_08915	99
CJ488_0998	838	*VgrG2*	CJE1141	56	A0W69_08980	98
			CJE1142	35	A0W69_08910	35
CJ488_0999	245		CJE1150	60	–	–
CJ488_1000	57		–	–	–	–
CJ488_1001	286		–	–	–	–
CJ488_1002	650		CJE1151	43	A0W69_08945	51
CJ488_1003	424		CJE1152	55	–	–
CJ488_1004	415		CJE1153	96	–	–

**TABLE 3:** *C. jejuni* strains containing CJIE3 by presence or absence of a T6SS.

	**No. of strains/total number of strains (%)**		
**Strain identity**	**CJIE3+**	**CJIE3−**	**T6SS-harbouring plasmid**
T6SS+	117/512 (22.85)	3/512 (0.59)	15/512 (2.93)
T6SS−	29/512 (5.66)	348/512 (67.97)	N/A
Total	146/512 (28.51)	351/512 (68.55)	15/512 (2.93)

*C. jejuni* Integrated Element 3 belongs to a large family of mobile genetic elements (MGEs) and, as observed in integrative and conjugative elements, could potentially operate horizontal transfer of DNA regions between bacterial species during extended periods of close proximity (Dobrindt et al., 2004; Johnson and Grossman, 2015). MGEs can also exist as PAIs; a large subset of integrative elements (>10 kb) carrying virulence genes, such as secretion systems and their cognate effectors (Jarvis et al., 1995; da Cruz Campos et al., 2020). Members of the order *Bacteroidales* can display three different “genetic architectures,” with two of these (GA1 and GA2) found on integrative conjugative elements (Coyne et al., 2016). CJIE3 shares sequence homology with proteins encoded on the *Campylobacter coli* RM2228 megaplasmid and 71-kb pathogenicity island HHGI1 of *Helicobacter hepaticus* ATCC51449, the latter possessing a T6SS (Fouts et al., 2005; Bartonickova et al., 2013). Interestingly, several *C. jejuni* megaplasmids also carry T6SS genes (Gunther et al., 2016; Marasini and Fakhr, 2016, 2017). Most recently, megaplasmids pCJDM202 and pCJDM67L from *C. jejuni* WP2-202 and OD2-67 strains, respectively, were found to harbour the T6SS cluster, along with the tetracycline resistance gene *tetO*, and T4SS conjugative DNA transfer systems (Marasini et al., 2020). The authors demonstrated that the presence of the T6SS on the megaplasmids contributed to enhanced haemolysis, suggested to support the survival of *C. jejuni* in retail meats (Marasini et al., 2020).

**FIGURE 2:**
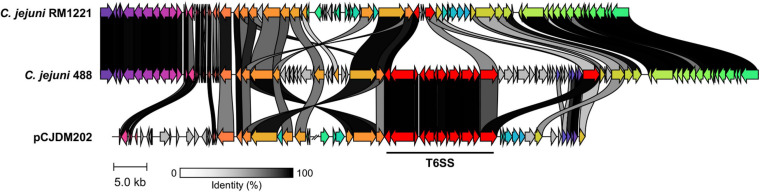
Comparative analyses of CJPI-1 (middle) from T6SS-positive *C. jejuni* 488 strain to CJIE3 from T6SS-negative strain RM1221 (top) and T6SS-positive megaplasmid pCJDM202 (bottom) from *C. jejuni* WP2-202 strain. Homologous genes across the three strains share the same arrow colour and are connected by shaded bands corresponding to sequence identity (black = 100%, white = 0%). Red arrows represent major T6SS components. Sequences found in only one strain possess a grey arrow. Only regions of the megaplasmid associated with the T6SS and CJIE3/CJPI-1 regions have been included. The figure was constructed using Clinker.

A newly re-sequenced and assembled genome of the T6SS-positive *C. jejuni* 488 strain was thus comparatively analysed against the genome of T6SS-negative *C. jejuni* RM1221 strain and T6SS-positive virulence megaplasmid pCJDM202 to investigate the genomic architecture and integration of the T6SS into the CJIE3 of *C. jejuni* (Figure 2). We propose to reclassify the T6SS-containing genomic island in the same chromosomal location as CJIE3 (between arginyl-tRNA-3 and *cje1156* in RM1221) as a new PAI-variant designated as CJPI-1. We observed that the ∼70 kb PAI is longer than the ∼50 kb CJIE3 of RM1221, containing an integrase/recombinase (*CJ488_0930*) gene (discussed below) and, like CJIE3, is located immediately adjacent to the chromosomal arginyl-tRNA (Table 4). The G + C% contents of both CJPI-1 and CJIE3 are lower than the average content of the 488 and RM1221 genomes, respectively, confirming the hypothesis that both inserted genetic elements could be considered as independently acquired. Furthermore, a direct repeat sequence designated as the attachment (*att*) sites, “TCCTCTTGAGCGCACCAT,” flanks both sides of the CJPI-1 and CJIE3 islands. Given the similarities between the integrated islands, CJPI-1 is most likely a derivative of CJIE3 that has undergone multiple recombination and/or genetic exchange events. We also discovered that only 30 proteins encoded in CJPI-1 share homology with those encoded in the CJIE3 of RM1221, highlighting differences in genetic composition (Table 2). Unlike CJIE3, CJPI-1 satisfies criteria commonly used to classify PAIs with the possession of the major T6SS components and putative effectors (discussed below). PAIs in other bacteria have also been found to carry T6SS clusters. Notably, HHGI-1 of *H. hepaticus* ATCC 51449 possesses a T6SS with a similar gene organisation to *C. jejuni* (Nano and Schmerk, 2007; Barker et al., 2009; Blondel et al., 2009; Bleumink-Pluym et al., 2013). In all, this data confirms that CJPI-1 can be considered as a *bona fide* PAI.

**TABLE 4:** Comparative overview of the sequence, gene content, and synteny characteristics of the CJPI-1 from *C. jejuni* 488 strain and CJIE3 from RM1221.

**Characteristics**	**CJPI-1 (488)**	**CJIE3 (RM1221)**
Length	∼70.3 kb	∼50.8 kb
CDS	77	62
Adjacent tRNA locus	Arginyl-tRNA	Arginyl-tRNA
Integrase locus tag	CJ488_0930c	CJE1094
G + C content	26.73%	26.62%
Genome G + C content	30.26%	30.31%
Flanking repeat sequence	TCCTCTTGAGCGCACCAT	TCCTCTTGAGCGCACCAT
Virulence factors	T6SS, putative effectors/toxins	N/A

Comparative analysis of the CJPI-1 to the virulence plasmid pCJDM202 also revealed striking genetic similarities (Figure 2). 35 genes in CJPI-1 matched in pCJDM202, including the T6SS and several genes also found in the CJIE3 (Table 2). Interestingly, the T6SS of CJPI-1 and pCJDM202 share 96% nucleotide similarity across the entire gene cluster. Collectively, this data suggests that CJPI-1 may be resulting from a recombination event of the CJIE3 and a T6SS-containing pCJMD202-like plasmid, leading to the acquisition and integration of the T6SS. However, further analyses are required to understand the genetic events leading to the acquisition of putative effectors (discussed below) which may have occurred through uptake events (mediated by prophages and plasmids conjugation).

### Functionally Predicted Proteins Encoded in CJPI-1

**FIGURE 3:**
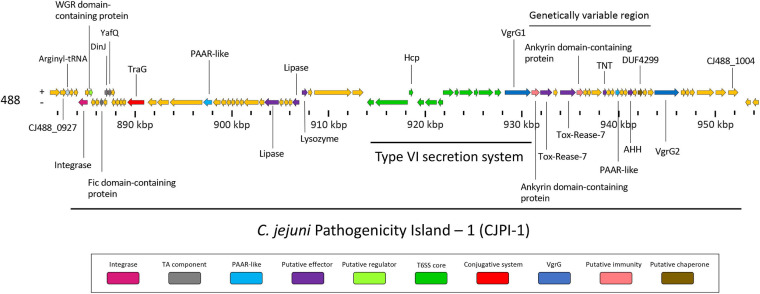
Genomic architecture of the PAI CJPI-1 in T6SS-positive *C. jejuni* 488 strain. Coloured coding domain sequences (CDS) represent proteins with inferred functions and are labelled. The scale under the CDS represents the nucleotide position [kilobase pair (kbp)] of the pathogenicity island in the genome of *C. jejuni* 488 strain. The location of the T6SS and genetically variable region are also denoted. The positive (+) symbol on the left-hand side indicates the sense strand in the genome of *C. jejuni* 488 strain, whilst the negative (–) symbol indicates the antisense strand. Genes sizes are not to scale. Genes are coloured according to predicted function: integrases are shaded magenta, regulators are light green, TA components are grey, conjugative systems are red, putative effectors are purple, PAAR-like proteins are light blue, T6SS components are green, VgrGs are dark blue, putative immunity proteins are pink, and putative chaperone proteins are olive.

Following the identification of this PAI-variant of CJIE3, we set out to bioinformatically investigate the genes encoded in CJPI-1 and assess their genomic context in relation to the PAI and/or T6SS operon. We identified several genes encoding for integrative elements, TA modules, and putative effectors (Table 2 and Figure 3).

#### Integrase

A putative integrase/recombinase (*CJ488_0930*) was identified within CJPI-1, possessing homology to the CJE1094 integrase (70% aa identity) and CJE1095 (26% aa identity) of RM1221, and A0W69_09480 (97% aa identity) from pCJDM202. The CJPI-1 integrase exhibits a Phage_Integrase (PF00589) and a tyrosine recombinase XerD (TIGR02225) domain and is likely a combination of CJE1094 and CJE1095 proteins. Both domains belong to the C-terminal catalytic domains of the DNA breaking-rejoining enzymes superfamily (cl00213). Proteins of this family catalyse recombination of DNA, possessing site-specific integration functions identified in chromosomes, plasmids, and phage genomes (Nash, 1996; Grainge and Jayaram, 1999; Huber and Waldor, 2002). It is predicted that the CJPI-1 and CJIE3 homologous integrases may be responsible for the horizontal transfer and chromosomal integration of the genomic islands to naïve strains at *att* sites (Santoriello et al., 2020).

#### Toxin-Antitoxin Modules

CJ488_0935, encoded in CJPI-1, was predicted to belong to the globular Fic/Doc (PF02661) family, harbouring the conserved Fic-motif H-x-F-x-[DE]-[AG]-N-[GK]-R, including the catalytic histidine residue which contributes to AMPylation activity (Sprenger et al., 2017; Veyron et al., 2018). Within our local database, 61 out of the 512 (11.91%) genomes were found to possess the protein CJ488_0935, of which 37 were T6SS-positive (60.66%) and 24 were T6SS-negative (39.34%). Of the T6SS-negative genomes, 12 out of the 24 were found to contain the CJIE3 (Supplementary Table 11). The protein CJE1100 from RM1221 also shares homology to CJ488_0935 (68% aa identity). Identified in a number of bacterial virulence factors (including Type III and IV secreted effectors), the AMPylation activity of Fic proteins have been demonstrated to catalyse the post-translational modifications of host proteins. Such activity leads to cytotoxicity of targetted host cells, as demonstrated in VopS from *Vibrio parahaemolyticus* and IbpA from *Haemophilus somnus* (Schmid et al., 2006; Worby et al., 2009; Yarbrough et al., 2009; Engel et al., 2012).

Fic domains can also be found as part of Type II TA toxin modules, as recently discovered in *Campylobacter fetus* (Goepfert et al., 2013; Sprenger et al., 2017). TA modules consist of a pair of antagonistic genes that encode for a stable toxin and adjacent, unstable antitoxin (Page and Peti, 2016). Many bacterial and archaeal chromosomes bear TA modules, with roles ranging from plasmid inheritance, MGE stability, growth arrest, and control to stress responses (Leplae et al., 2011; Page and Peti, 2016; Fraikin et al., 2020). Type II TA modules are the most widely studied TA systems in bacteria and have been identified to maintain and stabilise integrative elements, as well as involved to increase colonisation and virulence in pathogenic bacteria (Wozniak and Waldor, 2009; Leplae et al., 2011; Norton and Mulvey, 2012). TA modules have been characterised in *C. jejuni*, as observed in the pVir plasmids of 81-176 and IA3902 strains, and more recently in the YH002 strain isolated from retail beef liver (Shen et al., 2016; Ghatak et al., 2020).

Here, bioinformatic analysis inferred CJ488_0937 and CJ488_0938 as a putative Type II TA module, with the latter found to contain a predicted YafQ_toxin (PF15738) domain. YafQ toxins exhibit endoribonuclease activity and acts as the toxin component, with its activity inhibited by the cognate antitoxin DinJ (Motiejunaite et al., 2007; Prysak et al., 2009). Homologous proteins to CJ488_0938 were also found in RM1221 and pCJDM202, sharing 92 and 99% amino acid identity to CJE1103 and A0W69_09395, respectively. Within our local *C. jejuni* database, 95 out of the 512 (18.55%) genomes were found to possess the protein CJ488_0938, of which 64 were T6SS-positive (67.37%) and 31 were T6SS-negative (32.63%). Of the T6SS-negative genomes, 13 out of the 31 were found to contain the CJIE3 (Supplementary Table 11). The protein encoded by the upstream gene *CJ488_0937* (Figure 3) also shared an amino acid identity of 97% and 99% to proteins CJE1102 and A0W69_09400, respectively. CJ488_0937 does not possess any identifiable domains, therefore, we performed structural homology modelling of CJ488_0937 in the Phyre2 and I-Tasser servers using the amino acid sequence as a template (Zhang, 2008; Kelley et al., 2015). I-Tasser confidently identified the *Escherichia coli* DinJ-YafQ Type II TA complex (PDB: 4Q2U, Z-score: 1.60) as the most suitable candidate template for modelling, with an exclusive alignment to the DinJ antitoxin (PDB: 4Q2U_1) amino acid sequence. Further, both servers generated models with two predicted helix-turn-helix motifs suggesting, a DNA-binding function commonly identified in type II antitoxins (Aravind et al., 2005; Page and Peti, 2016). This is consistent with the DNA-binding ability and subsequent transcriptional autorepression activity of DinJ, the YafQ antitoxin (Ruangprasert et al., 2014). These elements suggest CJ488_0937 may be acting as the cognate DinJ antitoxin, with experimental confirmation warranted to validate these roles. To our knowledge, this is the first report of a DinJ-YafQ TA module in *Campylobacter* spp.

#### Conjugative Systems

The protein encoded by the gene *CJ488_0944* was predicted to contain a TraG N-terminal region (PF07916) domain and shares homology to proteins CJE1107 (92% aa identity) and A0W69_09385 (58% aa identity) from RM1221 and pCJDM202, respectively, with the latter annotated as a conjugation transfer protein TraG (Marasini and Fakhr, 2016). Within our local *C. jejuni* database, 93 out of the 512 (18.16%) genomes were found to possess the protein CJ488_0944 (average per genome = 1.04), of which 33 were T6SS-positive (35.48%) and 60 were T6SS-negative (64.52%). Of the T6SS-negative genomes, 5 out of the 60 were found to contain CJIE3 (Supplementary Table 11). The N-terminus of TraG is required for F pilus assembly; a long filament mediating the conjugative transfer of genetic material (Frost et al., 1994). Homologues of TraG and transfer coupling protein VirD4, a component of the *Agrobacterium tumefaciens* Type IVa secretion system (T4SSa), have been previously identified in the chromosomes of *C. jejuni* 81-176 and ATCC 43431, as well as in plasmids pCC31 and pTet (Batchelor et al., 2004; Poly et al., 2005; Chandran Darbari and Waksman, 2015). T4SS DNA conjugation systems were recently found harboured on megaplasmids in *C. jejuni* (Grohmann et al., 2018; Marasini et al., 2020). The T6SS-positive and TraG-containing megaplasmid pCJDM202 from *C. jejuni* WP2-202 strain was successfully transferred by conjugation to a T6SS-negative mutant NCTC11168 Nal^+^ recipient cell, with the *hcp* gene observed in the transconjugants, thus demonstrating a role in the transfer of T6SS-containing megaplasmids to transconjugants (Marasini et al., 2020).

#### Putative Regulator

CJ488_0932 was predicted to contain the nucleic acid binding domain WGR (smart00773), belonging to the WGR superfamily (cl01581), and shares homology to the protein A0W69_09470 (81% aa identity) from pCJDM202. Within our local *C. jejuni* database, 63 out of the 512 (12.30%) genomes were found to possess the protein CJ488_0932, of which 51 were T6SS-positive (80.95%) and 12 were T6SS-negative (19.05%) (Supplementary Table 11). WGR domains have been identified in poly(ADP-ribose) polymerases of eukaryotes, as well as in a molybdate metabolism regulator in *E. coli* and a number of predicted proteins (Citarelli et al., 2010); however, the precise function of the domain remains unclear.

#### Putative Effectors

##### Lipases

A number of T6SS lipase effectors have been previously described to target bacterial and eukaryotic membranes (Miyata et al., 2011; Russell et al., 2013; Jiang et al., 2014). In CJPI-1, two predicted lipase (Lipase class 3) domain-containing proteins (PF01764), CJ488_0957 and CJ488_0961, were identified upstream of the T6SS operon (Figure 3), with the former sharing homology to the proteins CJE1115 (64% aa identity) and A0W69_09355 (62% aa identity) from RM1221 and pCJDM202, respectively. Within our local *C. jejuni* database, 108 out of the 512 (21.09%) genomes were found to possess the protein CJ488_0957 (average per genome = 1.06), of which 98 were T6SS-positive (90.74%) and 10 were T6SS-negative (9.26%). Furthermore, 49 out of the 512 (9.57%) genomes were found to possess the protein CJ488_0961 (average per genome = 1.00), of which 45 were T6SS-positive (91.84%) and four were T6SS-negative (8.16%) (Supplementary Table 11). Previous PF01764 domain-containing proteins have been predicted as T4SS effectors in several bacteria targetting prokaryotic membranes, suggesting these two lipase-domain containing proteins belong to a larger family of hydrolysing effectors that can be delivered by several effector delivery systems (Sgro et al., 2019).

##### Lysozyme

A phage lysozyme-like protein, CJ488_0962 was also inferred in CJPI-1 and predicted to contain a Phage_lysozyme (PF00959) and autolysin/endolysin family (cd00737) domain. Within our local *C. jejuni* database, 96 out of the 512 (18.75%) genomes were found to possess the protein CJ488_0962 (average per genome = 1.02), of which 91 were T6SS-positive (94.79%) and five were T6SS-negative (5.21%) (Supplementary Table 11). PF00959 is described as a glycoside hydrolase, associated with bacteriophage enzymes that degrade bacterial peptidoglycan in the cell wall (El-Gebali et al., 2019). Several characterised bacteriophage endolysins have been demonstrated to exhibit lytic antibacterial activity, containing both domains predicted in CJ488_0962 (Li et al., 2016; Ding et al., 2020). Interestingly, a prevalence study identifying endolysins in phage genomes identified PF00959 as the most frequently detected domain amongst analysed phage endolysins predicted to target *Proteobacteria* (Fernandez-Ruiz et al., 2018).

### Diversity of Two VgrG Proteins Encoded in *C. jejuni* 488 Strain

**FIGURE 4:**
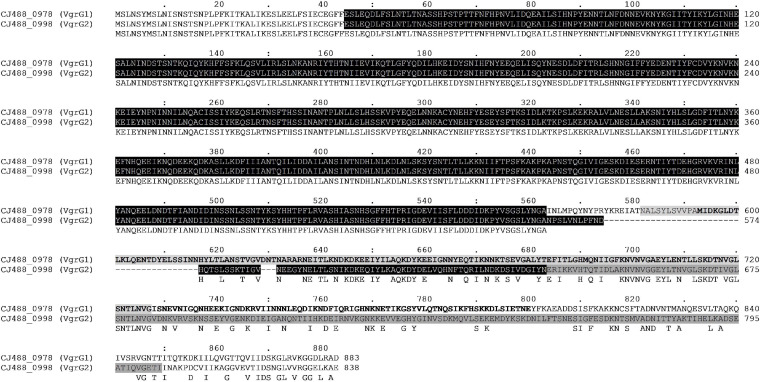
Full amino acid sequence alignment of the two VgrG homologues present in T6SS-positive *C. jejuni* 488 strain using ClustalO. Conserved residues found in both sequences correspond to letters in *uppercase* below the aligned sequences. Protein locus tag and amino acid position are provided for each respective sequence. The N-terminal VgrG domain (COG3501) in both sequences is highlighted black. The Jag domain (COG1847) is highlighted light grey for VgrG1, while letters in bold represent the Phyre2 structural homology match to the C-terminal lysozyme domain of Gp5 (PDB: 1K28). The Gp5_C domain (PF06715) is highlighted dark grey for VgrG2.

The protein VgrG is an essential component of the T6SS with roles including the promotion of the T6SS machinery assembly, the puncturing of target cells, and the delivery of effectors via their C-terminal domains (Pukatzki et al., 2007). Orphan *vgrG* genes can be located distally from their cognate T6SS structural operons; however, to date only one VgrG protein has been described in T6SS-containing *C. jejuni* (De Maayer et al., 2011; Bleumink-Pluym et al., 2013; Lopez et al., 2020). Here, we have identified two *vgrG* genes in the CJPI-1 of *C. jejuni* 488 (Figure 3), hereafter referred to as, *vgrG1* (*CJ488_0978*) and *vgrG2* (*CJ488_0998*) (Table 2 and Supplementary Table 2). Sequence alignment and identification of conserved and additional domains revealed a conserved N-terminal region possessing the VgrG domain (COG3501) and a region matching the superfamily VI_Rhs_Vgr (TIGR03361) (Figure 4). As observed in other bacteria, VgrG1 and VgrG2 differ in length, attributed to divergent C-terminal regions and associated domains (Hachani et al., 2011). The C-terminal region of VgrG1 (aa 564-883) shares an amino acid identity of 35% to the C-terminal region of VgrG2 (aa 564-838). Bioinformatic analysis revealed a Jag domain (COG1847, E-value: 0.008) in VgrG1, following the domain COG3501, which is potentially linked to RNA-binding. However, a structural homology search of the VgrG1 C-terminal region (aa 564-883) using Phyre2 matched to the C-terminal domain of the phage tail-lysozyme protein Gp5 (PDB: 1K28, Confidence: 100%). VgrG2 was found to contain a five superfamily/Gp5_C domain (PHA02596/PF06715) in its C-terminal region. The Gp5 C-terminal domain is commonly found in the bacteriophage T4 tail lysozyme protein Gp5 and VgrG proteins of bacteria, forming the membrane-puncturing β-helix structure of the spike proteins (Kanamaru et al., 2002; Pukatzki et al., 2007; El-Gebali et al., 2019). The C-termini Gp5 regions of some VgrG proteins may be also extended with catalytic domains (Hachani et al., 2011; Wettstadt et al., 2020). Furthermore, these extensions may also contribute to the recruitment of additional effectors (Flaugnatti et al., 2020).

A BLASTP-homology search was then conducted to assess the sequence identity of VgrG1 and VgrG2 against the NCBI Reference Protein database. The amino acid sequence of VgrG1 shares homology with the VgrG proteins of *C. coli* (WP_070241948.1–98.87% aa identity) and to Epsilonproteobacteria *Helicobacter* sp. MIT 11-5569 (WP_138109445.1–69.88% aa identity). Similarly, the amino acid sequence of VgrG2 shares homology with the VgrG proteins of *C. coli* (WP_072231509.1–99.76% aa identity) and to *Helicobacter* sp. MIT 11-5569 (WP_181881862.1–79.01% aa identity). The highly homologous matches suggest that these species too possess more than one VgrG protein with similar, if not identical domain architectures to VgrG1 and VgrG2, respectively. Further exploration is needed to assess whether both VgrG proteins exist in T6SS-containing *C. coli* and/or *Helicobacter* spp. genomes.

**FIGURE 5:**
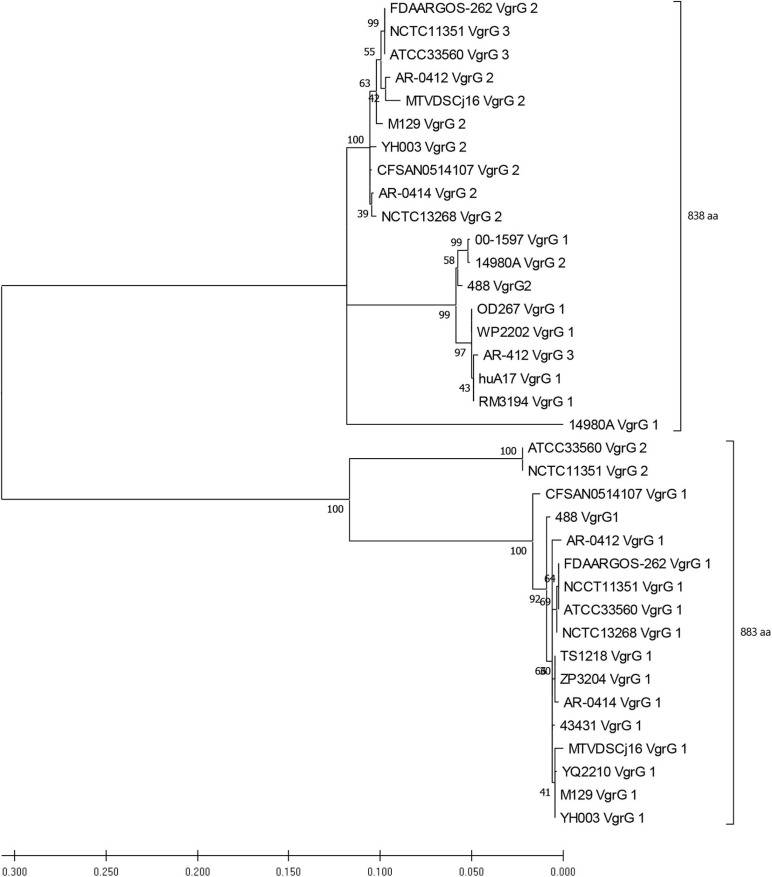
Phylogenetic tree derived from a Maximum likelihood analysis on a MUSCLE alignment of VgrG amino acid sequences from T6SS-positive *C. jejuni* of assembly level “complete” or higher (bootstrap *n* = 500, partial deletion). The value of each node is given. Top bracket = 838 amino acids (aa), bottom bracket = 883 aa.

Genomic analyses of T6SS-positive *C. jejuni* strains showed that a number of putative *vgrG* genes were located downstream of T6SS gene operons. A BLASTN search for *vgrG* detected 41 homologous genes in the T6SS-positive *C. jejuni* genomes (with assembly level “complete” or higher) (Supplementary Table 12). Interestingly, 1 of the 24 T6SS-positive “complete” *C. jejuni* genomes, IF1100, does not encode any *vgrG* gene, whereas 13 out of 24 encoded two or more. Phylogenetic analysis classified the VgrG proteins into two distinct clades, grouped with either VgrG1 or VgrG2 from *C. jejuni* 488 strain (Figure 5) (five VgrG amino acid sequences from genomes CJ017CC464, CJ018CCUA, and ZS007 were removed due to fragmented ORFs). A domain search using NCBI-CDD revealed that 28 of the 36 identified VgrG protein sequences contain an additional domain in the divergent C-terminus. Of which, 16 possessed the Jag (COG1847) domain, 12 possessed the five superfamily (PHA02596) domain, and eight possessed no identifiable domains (Supplementary Table 13). Collectively, this data suggests that two distinct VgrG proteins exist within the T6SS-positive *C. jejuni* isolates, with some strains bearing multiple VgrG proteins. It is predicted that isolates may exploit these different VgrG proteins in an interchangeable puncturing role in the spike complex, translocating specific effectors via interaction with the distinct C-terminal regions (Hachani et al., 2014; Jana and Salomon, 2019).

### Investigation of PAAR-Motif in *C. jejuni* T6SS-Positive and T6SS-Negative Genomes

**FIGURE 6:**
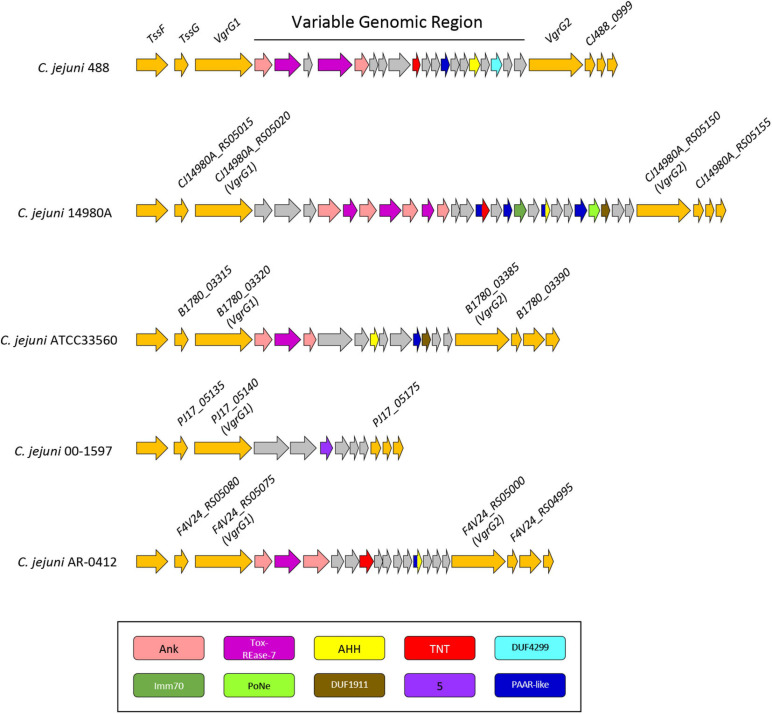
Genomic architecture of the genetically variable region downstream of the T6SS operon in five T6SS-positive *C. jejuni* strains; 488, 14980A, ATCC33560, 00-1597, and AR-0412. Genes found within the variable region possessing a domain by NCBI CDD (Marchler-Bauer et al., 2017) are coloured according to the domains identified: Ankyrin domains are pink, Tox-REase-7 are purple, AHH are yellow, TNT are red, DUF4299 are sky blue, Imm70 are dark green, PoNe are light green, DUF1911 are brown, five superfamily domain are lavender, and PAAR-like are dark blue. Variable region genes with no predicted function or identifiable domains are shaded grey and genes neighbouring the variable region are orange. Gene sizes are not to scale. The corresponding locus tags for selected genes is labelled above accordingly.

The T6SS puncturing structure is composed by a VgrG trimer further sharpened with a capping PAAR domain-containing protein tip (Wettstadt et al., 2020). To assess the prevalence of these “effector markers,” the amino acid sequences of representative proteins belonging to all classes of PAAR subgroups found in the superfamily CL21497 were aligned against a local protein database of *C. jejuni* genomes (Supplementary Data 1). We identified only PAAR4 (representative protein AGP36489.1 of *Sorangium cellulosum* So0157-2) which matched positively to 500 genomes. However, the results were considered not significant as the alignment exclusively occurred at the C-terminal S41-peptidase domain (PF03572) of the representative protein and not the N-terminal PAAR-motif. We hypothesised that compared to other bacteria, significant sequence divergence in *C. jejuni* PAAR genes might exist and thus homology-based searches may not identify PAAR proteins. Further iterative based analysis (data not shown) was able to predict a novel PAAR-like domain (∼ 125 amino acids) present in two proteins within CJPI-1, CJ488_0948, and CJ488_0990, with the latter found in the genetically variable region downstream of the T6SS operon (Figure 3). Multiple sequence alignments uncovered the novel domain possesses a series of conserved cysteine and histidine residues similar to the PAAR-like domain DUF4280 (Rigard et al., 2016) and PAAR-containing proteins (Shneider et al., 2013) and is present in a wide range of bacterial families. A number of the PAAR-like domain-containing proteins possess N- or C-terminal extensions harbouring characterised toxin domains (Figure 6), conferring a toxin translocation function to PAAR in addition its sharpening role (Shneider et al., 2013). The predicted novel PAAR-like domain-containing proteins in strain 488 possessed no other identifiable domains, confining a sole sharpening role to these proteins in this strain (Shneider et al., 2013). An alignment using the amino acid sequences of representative proteins for MIX clans I–V was also conducted; however returned no positive matches (Supplementary Table 7; Salomon et al., 2014).

### Putative T6SS-Associated Effectors Are Found in a Genetic Variable Region Downstream of the T6SS in CJPI-1

Genomic regions neighbouring *vgrG* genes often encode a number of hypothetical proteins, with many determined as T6SS effector and immunity proteins (De Maayer et al., 2011; Lopez et al., 2020). Analyses of T6SS-positive *C. jejuni* completely assembled genomes revealed a genetically variable region immediately downstream of the T6SS operon that did not share sequence identity to any ORFs found in the CJIE3 of strain RM1221. This region ranged from six genes in the 00-1597 strain, to 25 genes in the 14980A strain, located downstream of the main cluster gene *vgrG* and was commonly located upstream of the second orphan *vgrG* gene (Figure 6). Within this genetically variable region in 488, a number of ORFs between *vgrG1* and *vgrG2* were analysed using predictive programes (Figure 3). This resulted in the predicted identification of four putative effectors (CJ488_0980, CJ488_0982, CJ488_0988, and CJ488_0994), two ankyrin repeat domain-containing proteins (CJ488_0979 and CJ488_0983), and a DUF4299 family protein (CJ488_0996).

Among the putative effectors, proteins CJ488_0980 and CJ488_0982 contain predicted domains belonging to the restriction endonuclease family Tox-REase-7 (PF15649), whilst CJ488_0994 possesses a predicted domain belonging to the Tox-AHH HNH/ENDO VII superfamily nuclease (PF14412). Both domains belong to large toxin superfamilies with predicted functions as DNases/nucleases and are secreted by a wide range of polymorphic effector delivery systems, including the T6SS (Zhang et al., 2011, 2012). CJ488_0994 shares weak homology to the protein A0W69_08930 (41% aa identity) from pCJDM202, also predicted to possess a Tox-AHH domain. Interestingly, none of the other inferred putative effectors were found on pCJDM202 or CJIE3, suggesting separate genetic transfer events have mediated the acquisition of these putative T6SS-associated effectors.

**FIGURE 7:**
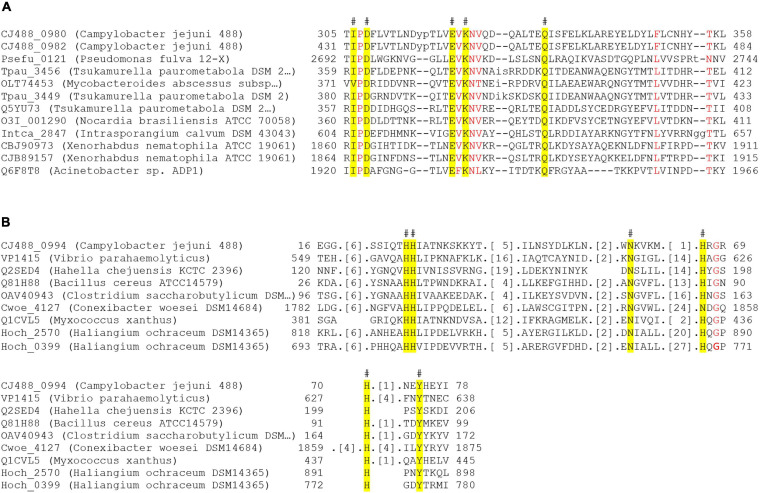
Partial amino acid sequence alignment of Tox-REase-7 and AHH domain-containing representative amino acids against putative effectors downstream of the T6SS from *C. jejuni* 488 strain. **(A)** Alignment of CJ488_0980 and CJ488_0982 against Tox-REase-7 representative amino acids. **(B)** Alignment of CJ488_0994 against AHH representative amino acids. Catalytic residues for each domain are highlighted in yellow, and the hash (#) indicates the position of the residue in the *C. jejuni* 488 proteins. Red letters indicate amino acids with greater than 90% homology across all sequences for the alignment. Protein locus tag, organism name, and amino acid position are to the left and either side of the respective sequences.

Both Tox-REase-7 domain-containing proteins, CJ488_0980 and CJ488_0982, contain the conserved catalytic residues IxD, ExK, and Q, and metal chelating site signature D-[EQ]xK, characteristic of REase-fold toxins, specifically Tox-REase-7 (Figure 7A). The AHH domain-containing protein CJ488_0994 also possesses the conserved catalytic residues HH, N, H, H, and Y, specific to the Tox-AHH fold/motif (Figure 7B; Zhang et al., 2012). Previous studies have successfully identified AHH domain-containing proteins as T6SS-associated effectors in both human and plant pathogenic bacterium, notably VP1415 from *V. parahaemolyticus*, therefore strengthening the prediction of CJ488_0994 as a T6SS effector (Figure 7B; Salomon et al., 2014; Santos et al., 2019). Within our local *C. jejuni* database, 88 out of the 512 (17.18%) genomes were identified to possess the protein CJ488_0980 (average per genome = 1.05), of which 87 were T6SS-positive (98.86%) and 1 was T6SS-negative (1.14%). The T6SS-negative genome was found to contain the CJIE3. Further, 100 out of 512 (19.53%) genomes were found to possess the protein CJ488_0994 (average per genome = 1.24), of which 95 were T6SS-positive (95%) and 5 were T6SS-negative (5%). Of the T6SS-negative genomes, 4 out of 5 were found to contain the CJIE3. Within the T6SS-positive population, 87 out of the 135 (64.44%) T6SS-positive genomes possessed the protein CJ488_0980 and 95 out of the 135 (70.37%) T6SS-positive genomes possessed the protein CJ488_0994 (Supplementary Table 11).

The final putative effector, CJ488_0988, was predicted to contain the C-terminal domain tuberculosis necrotising toxin (TNT) (PF14021) from the *Mycobacterium tuberculosis* protein CpnT (Sun et al., 2015). Within our local *C. jejuni* database, 83 out of the 512 (16.21%) genomes were found to possess the protein CJ488_0988 (average per genome = 1.12), of which 80 were T6SS-positive (96.39%) and three were T6SS-negative (3.61%). All of the T6SS-negative genomes were found to contain the CJIE3. Within the T6SS-positive population, 80 out of the 135 (59.26%) T6SS-positive genomes possessed the protein CJ488_0994 (Supplementary Table 11). TNT, characterised as a NAD^+^- and NAD(P)^+^-glycohydrolase, is transported to the cell surface of *M. tuberculosis* by CpnT and released by proteolytic cleavage, where inside the eukaryotic target cell it depletes cellular NAD^+^ inducing host-cell death (Danilchanka et al., 2014; Sun et al., 2015; Tak et al., 2019). The TNT domain of CJ488_0988 possesses the conserved residues Arg^5^, Arg^29^, and Gln^101^ (Arg^757^, Arg^780^, and Gln^822^ of TNT in *M. tuberculosis*), identified as the putative NAD^+^ binding pocket essential for NAD^+^ hydrolysis (Sun et al., 2015; Tak et al., 2019). The third tyrosine (Y) residue, Tyr^765^ in TNT of CpnT, was found to be replaced by a phenylalanine (F) residue, Phe^13^, in the TNT domain of CJ488_0988. Mutations of the Tyr^765^ residue were demonstrated to significantly reduce the catalytic activity of TNT yet did not eradicate its cytotoxic activity (Sun et al., 2015; Tak et al., 2019). Structural homology of CJ488_0988 using the Phyre2 server confidently identified the domain TNT (PDB: 4QLP/c4qlpB, Confidence: 99.6%) as the most suitable template for modelling with a sequence coverage of 98%, indicating a high sequence and structural similarity (Kelley et al., 2015). Several interbacterial Type VI effectors have also been characterised to exhibit NAD(P)^+^-glycohydrolase activity, inducing bacteriostasis in target cells (Whitney et al., 2015; Tang et al., 2018).

In bacteria, ankyrin repeat-containing proteins have been characterised to act as immunity proteins (ImmAnk) against decrosslinking enzymes and a wide range of T6SS-associated toxin domains, including Tox-AHH (Zhang et al., 2012; Lambert et al., 2015). Both predicted ankyrin repeat-containing proteins, CJ488_0979 and CJ488_0983, were found adjacent to the putative Tox-REase-7 effectors, CJ488_0980 and CJ488_0982, and are predicted to encode the cognate immunity proteins to the respective effectors, presenting two identical effector-immunity pairs. Immunity proteins for the remaining predicted effectors could not be determined using our *in silico* methods, suggesting they may play an anti-eukaryotic role and are not involved in interbacterial competition.

CJ488_0996 was predicted to contain the domain DUF4299 (PF14132) and shares homology to the protein A0W69_08920 (100%) from pCJDM2020. However, no further functional data was available. Within our local *C. jejuni* database, 127 out of the 512 (24.80%) genomes were found to possess the protein CJ488_0996 (average per genome = 1.06), of which 122 were T6SS-positive (96.06%) and five were T6SS-negative (3.94%) (Supplementary Table 11). The significantly high proportion of T6SS-positive genomes identified to possess CJ488_0996 suggests there is unknown link ensuring a strong conservation of both the T6SS and this DUF4299 domain-containing protein together. We speculate that the DUF4299 domain-containing protein may be playing an adaptor/chaperone protein role for the T6SS, as no identifiable toxin or effector domains could be determined (Liang et al., 2015; Bondage et al., 2016).

## Conclusion

The roles of T6SSs have been associated with interbacterial competition, host colonisation and virulence, as well as environmental survival. We have conducted in this study a comprehensive bioinformatic analysis to understand the genotypic T6SS organisation and its functional roles in *C. jejuni*. Using more than 500 publicly available genomes, we have identified co-occurrence of the T6SS and the integrative element CJIE3, confirming their association. Interestingly, genetic recombination with a T6SS-harbouring pCJDM202-like plasmid gives the potential for chromosomal integration. The analysis of our newly re-sequenced and assembled T6SS-positive 488 strain shows poor homology between the “T6SS-containing genomic island” and CJIE3, thus endorsing the reclassification of the former as a PAI termed CJPI-1. To note, Clark et al. (2018) make a similar observation when comparing the genomes and proteomes of four *C. jejuni* strains; however, here we present a comprehensive bioinformatics overview of the dynamic *C. jejuni* genome, its respective T6SS, and prediction of associated effectors (Clark et al., 2018). Two canonical VgrG proteins were identified within T6SS-positive *C. jejuni* genomes, as well as a wide range of predicted T6SS effectors and toxins, with some found in a genetically variable region downstream of the T6SS. Furthermore, we uncovered a putative DinJ-YafQ Type II TA module with predicted links to the stability of MGEs within *C. jejuni*. However, we cannot exclude from our analyses the possibility of these toxins to be recognised by the T6SS and exploited as effectors (Yadav et al., 2021). Collectively, these observations emphasise the diversity of genetic elements within *C. jejuni* strains, highlighting their contributions to bacterial survival in a wide range of hosts (i.e., chickens and humans) and in mediating competition in polymicrobial environments via multiple virulence factors.

Our data predicts the first T6SS-associated effectors in *C. jejuni* and identifies their putative functions as nucleases and a NAD^+^-glycohydrolase, based on their close proximity, high prevalence, and genomic context to the T6SS. Furthermore, the presence of putative anti-eukaryotic and anti-prokaryotic effectors suggests that *C. jejuni* encodes a multifunctional T6SS, as observed in other bacteria, that may have evolved during its evolutionary adaptation to host gastrointestinal tracts amongst polymicrobial communities (Miyata et al., 2013). Encoding a diverse repertoire of effectors, in close proximity to the T6SS operon, may allow for *C. jejuni* to secrete several effectors into prey cells and the surrounding milieu, overcoming bacterial competition and host defences to support its fitness.

This study also highlights that the acquisition of the T6SS and its related effectors into CJIE3 may have not occurred as a single event but rather upon multiple and independent genetic uptakes. Indeed, a small number of CJIE3-positive genomes were identified to possess some of the putative effectors in absence of a T6SS operon. This raises questions whether CJIE3-containing genomes once possessed a T6SS but was consequently lost through unknown events whilst retaining the putative effectors. Conversely, the effectors may have been associated to a number of pre-existing genes, some possibly as part of TA modules, subsequently repurposed as effectors upon successful acquisition of the T6SS into the genome via a plasmid, as previously mentioned. Interestingly, several T6SS-positive genomes were also identified to possess none of the putative effectors characterised in *C. jejuni* 488, suggesting they may instead harbour effector subsets that are yet to be discovered.

## Data Availability Statement

The original raw data used in this study are publicly available. This data can be found here: PRJEB41135. Publicly available datasets were analysed in this study. This data can be found here: https://www.ncbi.nlm. nih.gov.

## Author Contributions

OG and LR conceived the study. LR conducted the bioinformatic analysis and analysed the data. OG managed the study. LR, AH, and OG drafted the manuscript with contributions from JL, ZO, DX, AHMVV, and NC. All authors contributed to data interpretation.

## Conflict of Interest

The authors declare that the research was conducted in the absence of any commercial or financial relationships that could be construed as a potential conflict of interest.

## Supplementary Material

Supplementary Table 1 List of characterised T6SS effectors screened against the local *C. jejuni* protein database.

Supplementary Table 2 Domain and motif hits of predicted proteins in CJPI-1 using NCBI CDD-BLAST, Pfam, SMART, HmmScan, PROSITE, CDART, SUPERFAMILY, MOTIF, and InterPro.

Supplementary Table 3 Results of subcellular localisation, signal prediction and transmembrane helices prediction of CJPI-1 predicted proteins with inferred functions using Psortb, CELLO, SignalP, TMPred, and TMHMM.

Supplementary Table 4 Table of *C. jejuni* genomes “scaffold” level or higher under study with T6SS and CJIE3 analysis.

Supplementary Table 5 BLASTP-homology search for T6SS proteins in the local *Campylobacter* protein database.

Supplementary Table 6 Core T6SS proteins identified in the *C. jejuni* genomes under study.

Supplementary Table 7 List of MIX proteins screened against the local *C. jejuni* protein database.

Supplementary Table 8 BLASTN-homology search and analysis for *cje1094* in the local *Campylobacter* nucleotide database.

Supplementary Table 9 BLASTN-homology search and analysis for *cje1105* in the local *Campylobacter* nucleotide database.

Supplementary Table 10 BLASTN-homology search and analysis for *cje1153* in the local *Campylobacter* nucleotide database.

Supplementary Table 11 Analysis of the BLASTP-homology searches for CJPI-1 functionally predicted proteins in the local *C. jejuni* protein database.

Supplementary Table 12 BLASTN-homology search and analysis for *vgrG* genes in a local database of T6SS-positive *C. jejuni* genomes of assembly level “complete” or higher.

Supplementary Table 13 NCBI-CDD webtool results for VgrG proteins from T6SS-positive *C. jejuni* genomes of assembly level “complete” or higher.

Supplementary Data 1 PAAR amino acid sequences screened against the local *C. jejuni* protein database.

Supplementary Data 2 *C. jejuni* 488 strain assembled GenBank (.gbk) file.
