# Corrigendum: Bioinformatic Analysis of the *Campylobacter jejuni* Type VI Secretion System and Effector Prediction

**DOI:** 10.3389/fmicb.2021.793252

**Published:** 2021-11-16

**Authors:** Luca Robinson, Janie Liaw, Zahra Omole, Dong Xia, Arnoud H. M. van Vliet, Nicolae Corcionivoschi, Abderrahman Hachani, Ozan Gundogdu

**Affiliations:** ^1^Faculty of Infectious and Tropical Diseases, London School of Hygiene & Tropical Medicine, London, United Kingdom; ^2^Comparative Biomedical Sciences, Royal Veterinary College, London, United Kingdom; ^3^School of Veterinary Medicine, Faculty of Health and Medical Sciences, University of Surrey, Guildford, United Kingdom; ^4^Bacteriology Branch, Veterinary Sciences Division, Agri-Food and Biosciences Institute, Belfast, United Kingdom; ^5^Bioengineering of Animal Science Resources, Banat University of Agricultural Sciences and Veterinary Medicine – King Michael the I of Romania, Timisoara, Romania; ^6^The Peter Doherty Institute for Infection and Immunity, Department of Microbiology and Immunology, University of Melbourne, Melbourne, VIC, Australia

**Keywords:** *Campylobacter jejuni*, Type VI Secretion System, T6SS effectors, T6SS immunity proteins, toxin-antitoxin, pathogenicity island

In the original article, there was a mistake in Supplementary Data 2 (Data Sheet 2.ZIP) as published. The most up-to-date file was not used. The corrected file for Supplementary Data 2 has been updated in the original article.

The authors apologize for this error and state that this does not change the scientific conclusions of the article in any way. The original article has been updated.

## Publisher's Note

All claims expressed in this article are solely those of the authors and do not necessarily represent those of their affiliated organizations, or those of the publisher, the editors and the reviewers. Any product that may be evaluated in this article, or claim that may be made by its manufacturer, is not guaranteed or endorsed by the publisher.

