# Supplementary material for: Bioinformatic Analysis of the Campylobacter jejuni Type VI Secretion System and Effector Prediction
Source: Front Microbiol. 2021 Jun 29;12:694824. doi: 10.3389/fmicb.2021.694824 (PMC8285248; doi:10.3389/fmicb.2021.694824)
Supplement: Supplementary Table 10 — BLASTN-homology search and analysis for cje1153 in the local Campylobacter nucleotide database. [file Data_Sheet_1.pdf]

Supplementary Material

1 Supplementary Tables

Table S1: List of characterised T6SS cargo effectors screened against the local *C. jejuni* protein database.

Species/Strain	Gene ID	Protein ID	Protein name	Reference
Pseudomonas aeruginosa PAO1	PA1844	AAG05233.1	Tse1	(Hood et al., 2010)
Pseudomonas aeruginosa PAO1	PA2702	AAG06090.1	Tse2	(Hood et al., 2010)
Pseudomonas aeruginosa PAO1	PA3484	AAG06872.1	Tse3 (Tge1)	(Hood et al., 2010)
Pseudomonas aeruginosa PAO1	PA2774	AAG06162.1	Tse4	(Whitney et al., 2014)
Pseudomonas aeruginosa PAO1	PA2684	NP_251374.1	Tse5 (RhsP1)	(Whitney et al., 2014)
Pseudomonas aeruginosa PAO1	PA0093	AAG03483.1	Tse6	(Whitney et al., 2014)
Pseudomonas aeruginosa PAO1	PA0099	AAG03489.1	Tse7	(Hachani et al., 2014)
Pseudomonas aeruginosa PAO1	PA2374	AAG05762.1	TseF	(Lin et al., 2017)
Pseudomonas aeruginosa PAO1	PA3487	NP_252177.1	PldA (Tle5a)	(Russell et al., 2013)
Pseudomonas aeruginosa PAO1	PA5089	AAG08474.1	PldB (Tle5b)	(Russell et al., 2013)
Serratia marcescens	SMA2261	WP_025303293.1	Ssp1	(English et al., 2012)
Serratia marcescens	SMA2264	WP_089196535.1	Ssp2 (Tae4)	(English et al., 2012)

Serratia marcescens	SMA1112	WP_025302370.1	Ssp3 (Tfe1)	(Fritsch et al., 2013)
Serratia marcescens	SMA3980	WP_025304642.1	Ssp4	(Fritsch et al., 2013)
Serratia marcescens	SMA4628	WP_025305124.1	Ssp5	(Fritsch et al., 2013)
Serratia marcescens	SMA4673	WP_025305162.1	Ssp6	(Fritsch et al., 2013)
Vibrio cholerae O1 biovar El Tor str. N16961	VCA0020	AAF95934.1	VasX	(Miyata et al., 2011)
Burkholderia thailandensis E264	BTH_I0068	ABD38716.1	Tae2	(Russell et al., 2012)
Salmonella enterica subsp. enterica serovar Typhimurium str. LT2	STM0277	AAL19234.1	Tae4	(Sana et al., 2016)
Serratia marcescens subsp. marcescens Db11	SMDB11_1083	CDG11658.1	Tfe2	(Trunk et al., 2018)
Pseudomonas protegens Pf-5	PFL_3037	AAY92307.1	Tge2	(Whitney et al., 2013)
Dickeya dadantii 3937	Dda3937_02773	ADM99131.1	RhsA	(Koskiniemi et al., 2013)
Agrobacterium fabrum str. C58	ATU4350	AAK89090.1	Tde1	(Ma et al., 2014)
Agrobacterium fabrum str. C58	ATU3640	AAK89757.1	Tde2	(Ma et al., 2014)
Pseudomonas protegens Pf-5	PFL_6209	AAY95397.1	Tne2	(Tang et al., 2018)
Burkholderia thailandensis E264	BTH_I2698	ABC38949.1	Tle1	(Russell et al., 2013)

Vibrio cholerae O1 biovar El Tor str. N16961	VC_1418	AAF94575.1	Tle2 (TleL)	(Russell et al., 2013)
Pseudomonas aeruginosa PAO1	PA0260	AAG03649.1	Tle3	(Russell et al., 2013)
Pseudomonas aeruginosa PAO1	PA1510	AAG04899.1	TplE	(Jiang et al., 2016)
Pseudomonas putida KT2440	PP3108	AAN68716.1	Tke2	(Bernal et al., 2017)
Pseudomonas aeruginosa PAO1	PA3290	AAG06678.1	Tle1	(Russell et al., 2013)
Vibrio parahaemolyticus	B5C30_RS14465	WP_029857615.1	v12_14465	(Jana et al., 2019)
Yersinia pseudotuberculosis YPIII	YPK_0954	ACA67255	Tce1	(Song et al., 2020)
Vibrio parahaemolyticus BB22OP	VPBB_RS15030	WP_015297525.1	Tme1	(Fridman et al., 2020)
Vibrio parahaemolyticus T9109	PO79_RS05910	WP_047706523.1	Tme2	(Fridman et al., 2020)
Salmonella Typhi str. Ty2	t0489	AAO68195.1	Tae3	(Russell et al., 2012)
Salmonella Typhimurium str. 14028S	STM14_0336	ACY86862.1	Tlde1	(Sibinelli-Sousa et al., 2020)
Acinetobacter baylyi ADP1	ACIAD0053	CAG67035.1	Tpe1	(Ringel et al., 2017)
Burkholderia cenocepacia H111	I35_7839	CDN65395.1	DddA	(Mok et al., 2020)
Serratia proteamaculans 568	Spro_3017	WP_012145739.1	Tre1	(Ting et al., 2018)

Table S2: Domain and motif hits of predicted proteins in CJPI-1 using NCBI CDD-BLAST, Pfam, SMART, HmmScan, PROSITE, CDART, SUPERFAMILY, MOTIF and InterPro.

Protein ID	NCBI-CDD	Pfam	SMART	HmmScan	PROSITE	CDART	SUPERFAMILY	MOTIF	InterPro
CJ488_0928			Uncharacterized protein						
CJ488_0929			Uncharacterized protein					pfam03029, ATP_bind_1, Conserved hypothetical ATP binding protein	
CJ488_0930	DNA_BRE_C (cd00397)<br><br>Tyrosine Recombinase<br><br>(TIGR02225)<br><br>Phage_Integrase<br><br>(pfam00589)	Phage_Integrase	Phage_Integrase	Phage<br><br>Integrase	Tyr<br><br>recombinase	Tyrosine Recombinase<br><br>Integrase	DNA breaking-rejoining enzymes	TIGR02225, Tyrosine recombinase XerD<br><br>pfam00589, Phage integrase	Integrase, catalytic domain<br><br>IPR002104

CJ488_0931			Mobilization protein						
CJ488_0932	Proposed nucleic acid binding domain<br><br>(smart00773)		Polymerase,<br><br>WGR domain SM000773			WGR domain-containing protein	WGR domain-like	smart00773, WGR, Proposed nucleic acid binding domain	
CJ488_0933			Uncharacterized protein						
CJ488_0934			Uncharacterized protein						
CJ488_0935	Fic/DOC family<br><br>(pfam02661)	Fic/DOC family	Fic domain<br><br>containing protein	Fic/DOC family	Fido domain containing protein	Fic family protein	Fic-like	Doc, Prophage maintenance system killer protein<br><br>pfam02661, Fic	Fido domain<br><br>IPR003812
CJ488_0936			DNA_primase_I rg						

CJ488_0937			Uncharacterized protein						
CJ488_0938	Bacterial toxin of type II toxin-antitoxin system, YafQ<br><br>(pfam15738)	Bacterial toxin of type II toxin-antitoxin system, YafQ	type II toxin-antitoxin system YafQ family toxin	Bacterial toxin of type II toxin-antitoxin system, YafQ		Type II toxin-antitoxin system RelE/ParE family toxin	RelE-like	pfam15738, YafQ_toxin, Bacterial toxin of type II toxin-antitoxin system	Toxin-antitoxin system, YafQ-like toxin<br><br>IPR004386
CJ488_0939			Uncharacterized protein						
CJ488_0940			Uncharacterized protein						
CJ488_0941			Uncharacterized protein						

CJ488_0942			Uncharacterized protein						
CJ488_0943			Uncharacterized protein						
CJ488_0944	TraG-like protein - TraG protein is known to be essential for DNA transfer in the process of conjugation<br><br>(pfam07916)	TraG-like protein, N-terminal region	TraG_N containing protein	TraG-like protein, N-terminal region		Conjugal transfer protein TraG		pfam07916, TraG_N	TraG-like, N-terminal<br><br>IPR012931
CJ488_0945			Uncharacterized protein						
CJ488_0946			Hypothetical protein						

CJ488_0947			Hypothetical protein						
CJ488_0948	DNA polymerase III PolC<br><br>(PRK00448)		Uncharacterized protein			PolC-type DNA polymerase III		PRK00448, polC, DNA polymerase III	
CJ488_0949			Uncharacterized protein						
CJ488_0950			Hypothetical protein						
CJ488_0951			Uncharacterized protein						
CJ488_0952			Hypothetical protein						

CJ488_0953			Hypothetical protein						
CJ488_0954			Hypothetical protein						
CJ488_0955			Uncharacterized protein						
CJ488_0956			Uncharacterized protein						
CJ488_0957	Lipase (class 3)<br>(pfam01764)		hypothetical protein		Lipase serine active site	hypothetical protein	alpha/beta-Hydrolases	pfam01764, Lipase_3	Protein of unknown function DUF2974,<br>(IPR024499)<br>Alpha/Beta hydrolase fold,<br>(IPR029058)

CJ488_0958			hypothetical protein						
CJ488_0959			hypothetical protein						
CJ488_0960			Uncharacterized protein						
CJ488_0961	Lipase (class 3)<br><br>(pfam01764)	Lipase (class 3)	hypothetical protein<br><br>(Lipase_3 domain containing protein)	Lipase (class 3)		hypothetical protein	alpha/beta-Hydrolases	cd00519, Lipase_3	Alpha/Beta hydrolase fold,<br><br>(IPR029058)
CJ488_0962	endolysin and autolysin - cd00737	Phage lysozyme	Phage_lysozyme containing protein	Phage lysozyme		lytic transglycosylase	Lysozyme	cd00737, lyz_endolysin_autolysin	Lysozyme-like domain superfamily<br><br>(IPR023346)<br><br>Endolysin/

								COG3772, Phage-related lysozyme	autolysin<br><br>(IPR033907)
CJ488_0963			Uncharacterized protein						
CJ488_0964			hypothetical protein					cd01059, CCC1_like	
CJ488_0965			hypothetical protein						
CJ488_0966	type VI secretion system FHA domain protein<br><br>(TIGR03354)	FHA domain	Uncharacterized protein<br><br>(FHA domain containing protein)	FHA domain		type VI secretion system-associated FHA domain protein TagH	SMAD/FHA domain	type VI secretion system FHA domain protein	
CJ488_0967	Type VI secretion protein IcmF	ImcF-related N-terminal domain	type VI secretion system membrane subunit TssM	ImcF-related N-terminal domain		type VI secretion system membrane subunit TssM	P-loop containing nucleoside triphosphate hydrolases	type VI secretion protein IcmF	

CJ488_0968	type VI secretion system effector, Hcp1	Type VI secretion system effector, Hcp	type VI secretion system tube protein Hcp	Type VI secretion system effector, Hcp		type VI secretion system tube protein Hcp	Hcp1-like	Type VI protein secretion system component Hcp	
CJ488_0969	Type VI secretion system protein DotU	Type VI secretion system protein DotU	DotU family type IV/VI secretion system protein	Type VI secretion system protein DotU		DotU family type IV/VI secretion system protein		Type VI secretion system protein DotU	
CJ488_0970	Bacterial Type VI secretion protein ImpJ, VasE	Bacterial Type VI secretion, VC_A0110, EvfL, ImpJ, VasE	TssK	Bacterial Type VI secretion, VC_A0110, EvfL, ImpJ, VasE		type VI secretion system baseplate subunit TssK		T6SS_VasE, Bacterial Type VI secretion	
CJ488_0971	Type VI secretion lipoprotein, VasD	Type VI secretion lipoprotein, VasD, EvfM, TssJ, VC_A0113	Lipoprotein, putative	Type VI secretion lipoprotein, VasD, EvfM, TssJ, VC_A0113	Prokaryotic membrane lipoprotein lipid attachment site profile	type VI secretion system lipoprotein TssJ		Type VI secretion lipoprotein, VasD	

CJ488_0972	Type VI secretion protein, VasJ	Type VI secretion, EvfE, EvfF, ImpA, BimE, VC_A0119, VasJ	Uncharacterized protein<br><br>(VasJ domain containing protein)	Type VI secretion, EvfE, EvfF, ImpA, BimE, VC_A0119, VasJ		type VI secretion system VasJ domain protein		T6SS_VasJ, Type VI secretion	
CJ488_0973	Type VI secretion system, VipA	Type VI secretion system, VipA, VC_A0107 or Hcp2	TssB	Type VI secretion system, VipA, VC_A0107 or Hcp2		type VI secretion system contractile sheath small subunit		Type VI secretion system, VipA	
CJ488_0974	type VI secretion protein, VipB	Type VI secretion protein, EvpB/VC_A0108, tail sheath	Type VI secretion system contractile sheath large subunit	Type VI secretion protein, EvpB/VC_A0108, tail sheath		type VI secretion system contractile sheath large subunit		VipB, Type VI secretion protein	
CJ488_0975	type VI secretion system lysozyme-like protein	Gene 25-like lysozyme	Tgh104	Gene 25-like lysozyme		GPW/gp25 family protein	gpW/gp25-like	type VI secretion system lysozyme-like protein	

CJ488_0976	Type VI secretion system, TssF	Type VI secretion system, TssF	Type VI secretion protein<br><br>(TssF domain containing protein)	Type VI secretion system, TssF		type VI secretion system baseplate subunit TssF		Type VI secretion system, TssF	
CJ488_0977	Type VI secretion, TssG	Type VI secretion, TssG	TssG	Type VI secretion, TssG		type VI secretion system baseplate subunit TssG		Type VI secretion, TssG	
CJ488_0978	type VI secretion system Vgr family protein<br><br>+<br><br>Jag domain	Phage late control gene D protein (GPD)	Phage_GPD domain containing protein	Phage late control gene D protein (GPD)		type VI secretion system tip protein VgrG	Phage tail proteins	type VI secretion system Vgr family protein	
CJ488_0979	Ankyrin repeat-	Ankyrin repeats (3 copies)	Ankyrin domain containing protein	Ankyrin repeats (3 copies)	Ankyrin repeat region	ankyrin repeat domain-	Ankyrin repeat	ANK, ankyrin repeats	Ankyrin repeat-

	containing protein<br><br>(pfam12796)				circular profile	containing protein			containing domain<br><br>(IPR020683)
CJ488_0980	Tox-REase-7 domain containing protein<br><br>(pfam15649)	Restriction endonuclease fold toxin 7	Tox-REase-7 domain containing protein	Restriction endonuclease fold toxin 7		Hypothetical protein		Tox-REase-7, Restriction endonuclease fold toxin 7	Tox-REase-7 domain<br><br>(IPR028903)
CJ488_0981			Hypothetical protein				Ankyrin repeat		Ankyrin repeat-containing domain<br><br>(IPR020683)
CJ488_0982	Tox-REase-7 domain containing protein<br><br>(pfam15649)	Restriction endonuclease fold toxin 7	Hypothetical protein	Restriction endonuclease fold toxin 7		Hypothetical protein		Tox-REase-7, Restriction endonuclease fold toxin 7	Tox-REase-7 domain<br><br>(IPR028903)

CJ488_0983	Ankyrin-like protein<br><br>(PHA03095)		Ankyrin domain containing protein		Ankyrin repeat region circular profile	ankyrin repeat domain-containing protein	Ankyrin repeat	ANK, ankyrin repeats	Ankyrin repeat-containing domain<br><br>(IPR020683)
CJ488_0984			Hypothetical protein						
CJ488_0985			Tgh071						
CJ488_0986			Uncharacterized protein						
CJ488_0987			Hypothetical protein						
CJ488_0988	Tuberculosis necrotizing toxin	Tuberculosis necrotizing toxin	TNT domain containing protein	Tuberculosis necrotizing toxin		DUF4237 domain-		pfam14021, TNT, Tuberculosis	Tuberculosis necrotizing toxin

	(pfam14021)					containing protein		necrotizing toxin	(IPR025331)
CJ488_0989			hypothetical protein						
CJ488_0990			hypothetical protein						
CJ488_0991	Bacterial toxin 24<br><br>(pfam15529)		hypothetical protein			Hypothetical protein		pfam15529, Ntox24, Bacterial toxin 24	
CJ488_0992			Uncharacterized protein						
CJ488_0993			hypothetical protein						
CJ488_0994	A nuclease family of the HNH/ENDO VII	A nuclease family of the HNH/ENDO VII superfamily	AHH-nuclease domain	A nuclease family of the HNH/ENDO VII		Hypothetical protein		pfam14412, AHH, A nuclease family of the	

	superfamily with conserved AHH<br><br>(pfam14412)	with conserved AHH	containing protein	superfamily with conserved AHH				HNH/ENDO VII superfamily with conserved AHH	
CJ488_0995			Uncharacterized protein						
CJ488_0996	Domain of unknown function (DUF4299)<br><br>(pfam14132)	Domain of unknown function (DUF4299)	Uncharacterized protein<br><br>(DUF4299 domain containing protein)	Domain of unknown function (DUF4299)		DUF4299 family protein		pfam14132, DUF4299	
CJ488_0997			Uncharacterized protein						
CJ488_0998	type VI secretion system Vgr family protein	Phage late control gene D protein (GPD)	Phage_GPD domain containing protein	Phage late control gene D protein (GPD)		type VI secretion system tip protein VgrG	Phage tail proteins	type VI secretion system Vgr family protein	

	+ baseplate hub subunit and tail lysozyme, 5								
CJ488_0999			Hypothetical protein						
CJ488_1000			Hypothetical protein						
CJ488_1001			Tgh113						
CJ488_1002			hypothetical protein						
CJ488_1003			hypothetical protein						
CJ488_1004			Uncharacterized protein						

Table S3: Results of subcellular localisation, signal prediction and transmembrane helices prediction of CJPI-1 predicted proteins with inferred functions using Psortb, CELLO, SignalP, TMPred and TMHMM.

488 Protein ID	TMHMM	TMPred	SignalP	CELLO	Psortb
CJ488_0930	0	0	No	Cytoplasmic	Cytoplasmic
CJ488_0932	0	0	No	Extracellular	Unknown
CJ488_0935	0	0	No	Cytoplasmic<br>Extracellular	Unknown
CJ488_0938	0	0	No	Cytoplasmic	Unknown
CJ488_0944	5	8	Yes (Sec/SPI)	Membrane	Cytoplasmic-Membrane
CJ488_0957	0	1	No	Cytoplasmic	Cytoplasmic
CJ488_0961	0	1	No	Cytoplasmic<br>Extracellular	Unknown
CJ488_0962	0	0	No	Extracellular	Unknown

CJ488_0966	0	0	No	Cytoplasmic	Cytoplasmic
CJ488_0967	3	3	No	Extracellular	Cytoplasmic-Membrane
CJ488_0968	0	0	No	Membrane	Extracellular
CJ488_0969	1	1	No	Cytoplasmic	Cytoplasmic
CJ488_0970	0	0	No	Cytoplasmic	Cytoplasmic
CJ488_0971	0	1	Yes (Sec/SPII)<br><br>Lipoprotein signal peptide	Membrane	Unknown
CJ488_0972	0	0	No	Cytoplasmic	Cytoplasmic
CJ488_0973	0	0	No	Cytoplasmic	Cytoplasmic
CJ488_0974	0	0	No	Cytoplasmic	Cytoplasmic
CJ488_0975	0	0	No	Cytoplasmic	Cytoplasmic

CJ488_0976	0	1	No	Cytoplasmic	Cytoplasmic
CJ488_0977	0	2	No	Cytoplasmic	Cytoplasmic
CJ488_0978	0	0	No	Extracellular	Cytoplasmic
CJ488_0979	0	0	No	Cytoplasmic	Cytoplasmic
CJ488_0980	0	0	No	Cytoplasmic	Cytoplasmic
CJ488_0982	0	0	No	Cytoplasmic	Cytoplasmic
CJ488_0983	0	0	No	Cytoplasmic	Cytoplasmic
CJ488_0988	0	0	No	Cytoplasmic	Cytoplasmic
CJ488_0994	0	0	No	Cytoplasmic<br>Extracellular	Cytoplasmic
CJ488_0996	0	0	No	Cytoplasmic	Unknown

CJ488_0998	0	0	No	Extracellular	Cytoplasmic

Table S7: List of MIX proteins screened against the local *C. jejuni* protein database.

**Species/Strain**	**Gene ID**	**Protein ID**	**MIX clan**	**Reference**
Vibrio parahaemolyticus RIMD 2210633	VP1388	BAC59651.1	MIX clan 1 motif	(Salomon et al., 2014)
Vibrio campbellii ATCC BAA-1116	VIBHAR_03070	ABU72020.1	MIX clan 2 motif	(Salomon et al., 2014)
Burkholderia thailandensis E264	BTH_I2691	ABC38088.1	MIX clan 3 motif	(Salomon et al., 2014)
Vibrio cholerae O1 biovar El Tor str. N16961	VC_A0020	AAF95934.1	MIX clan 4 motif	(Salomon et al., 2014)
Marinomonas sp. MWYL1	Mmwyl1_0527	ABR69463.1	MIX clan 5 motif	(Salomon et al., 2014)

Supplementary References

Bernal, P., Allsopp, L. P., Filloux, A., and Llamas, M. A. (2017). The Pseudomonas putida T6SS is a plant warden against phytopathogens. *ISME J.* 11, 972–987. doi:10.1038/ismej.2016.169.

English, G., Trunk, K., Rao, V. A., Srikannathasan, V., Hunter, W. N., and Coulthurst, S. J. (2012). New secreted toxins and immunity proteins encoded within the Type VI secretion system gene cluster of *Serratia marcescens*. *Mol. Microbiol.* 86, 921–936. doi:10.1111/mmi.12028.

Fridman, C. M., Keppel, K., Gerlic, M., Bosis, E., and Salomon, D. (2020). A comparative genomics methodology reveals a widespread family of membrane-disrupting T6SS effectors. *Nat. Commun.* 11, 1–14. doi:10.1038/s41467-020-14951-4.

Fritsch, M. J., Trunk, K., Diniz, J. A., Guo, M., Trost, M., and Coulthurst, S. J. (2013). Proteomic identification of novel secreted antibacterial toxins of the Serratia marcescens Type VI secretion system. *Mol. Cell. Proteomics* 12, 2735–2749. doi:10.1074/mcp.M113.030502.

Hachani, A., Allsopp, L. P., Oduko, Y., and Filloux, A. (2014). The VgrG proteins are “à la carte” delivery systems for bacterial type VI effectors. *J. Biol. Chem.* 289, 17872–17884. doi:10.1074/jbc.M114.563429.

Hood, R. D., Singh, P., Hsu, F. S., Güvener, T., Carl, M. A., Trinidad, R. R. S., et al. (2010). A Type VI Secretion System of Pseudomonas aeruginosa Targets a Toxin to Bacteria. *Cell Host Microbe* 7, 25–37. doi:10.1016/j.chom.2009.12.007.

Jana, B., Fridman, C. M., Bosis, E., and Salomon, D. (2019). A modular effector with a DNase domain and a marker for T6SS substrates. *Nat. Commun.* 10, 1–12. doi:10.1038/s41467-019-11546-6.

Jiang, F., Wang, X., Wang, B., Chen, L., Zhao, Z., Waterfield, N. R., et al. (2016). The Pseudomonas aeruginosa Type VI Secretion PGAP1-like Effector Induces Host Autophagy by Activating Endoplasmic Reticulum Stress. *Cell Rep.* 16, 1502–1509. doi:10.1016/j.celrep.2016.07.012.

Koskiniemi, S., Lamoureux, J. G., Nikolakakis, K. C., De Roodenbeke, C. T. K., Kaplan, M. D., Low, D. A., et al. (2013). Rhs proteins from diverse bacteria mediate intercellular competition. *Proc. Natl. Acad. Sci. U. S. A.* 110, 7032–7037. doi:10.1073/pnas.1300627110.

Lin, J., Zhang, W., Cheng, J., Yang, X., Zhu, K., Wang, Y., et al. (2017). A Pseudomonas T6SS effector recruits PQS-containing outer membrane vesicles for iron acquisition. *Nat. Commun.* 8, 1–12. doi:10.1038/ncomms14888.

Ma, L. S., Hachani, A., Lin, J. S., Filloux, A., and Lai, E. M. (2014). Agrobacterium tumefaciens deploys a superfamily of type VI secretion DNase effectors as weapons for interbacterial competition in planta. *Cell Host Microbe* 16, 94–104. doi:10.1016/j.chom.2014.06.002.

Miyata, S. T., Kitaoka, M., Brooks, T. M., McAuley, S. B., and Pukatzki, S. (2011). Vibrio cholerae requires the type VI secretion system virulence factor vasx to kill dictyostelium discoideum. *Infect. Immun.* 79, 2941–2949. doi:10.1128/IAI.01266-10.

Mok, B. Y., de Moraes, M. H., Zeng, J., Bosch, D. E., Kotrys, A. V., Raguram, A., et al. (2020). A bacterial cytidine deaminase toxin enables CRISPR-free mitochondrial base editing. *Nature* 583, 631–637. doi:10.1038/s41586-020-2477-4.

Ringel, P. D., Hu, D., and Basler, M. (2017). The Role of Type VI Secretion System Effectors in Target Cell Lysis and Subsequent Horizontal Gene Transfer. *Cell Rep.* 21, 3927–3940. doi:10.1016/j.celrep.2017.12.020.

Russell, A. B., Leroux, M., Hathazi, K., Agnello, D. M., Ishikawa, T., Wiggins, P. A., et al. (2013). Diverse type VI secretion phospholipases are functionally plastic antibacterial effectors. *Nature* 496, 508–512. doi:10.1038/nature12074.

Russell, A. B., Singh, P., Brittnacher, M., Bui, N. K., Hood, R. D., Carl, M. A., et al. (2012). A widespread bacterial type VI secretion effector superfamily identified using a heuristic approach. *Cell Host Microbe* 11, 538–549. doi:10.1016/j.chom.2012.04.007.

Russell, A. B., Singh, P., Brittnacher, M., Bui, N. K., Hood, R. D., Carl, M. A., et al. (2012). A widespread bacterial type VI secretion effector superfamily identified using a heuristic approach. *Cell Host Microbe* 11, 538–549. doi:10.1016/j.chom.2012.04.007.

Salomon, D., Kinch, L. N., Trudgian, D. C., Guo, X., Klimko, J. A., Grishin, N. V., et al. (2014). Marker for type VI secretion system effectors. *Proc. Natl. Acad. Sci. U. S. A.* 111, 9271–9276. doi:10.1073/pnas.1406110111.

Sana, T. G., Flaugnatti, N., Lugo, K. A., Lam, L. H., Jacobson, A., Baylot, V., et al. (2016). Salmonella Typhimurium utilizes a T6SS-mediated antibacterial weapon to establish in the host gut. *Proc. Natl. Acad. Sci. U. S. A.* 113, E5044–E5051. doi:10.1073/pnas.1608858113.

Sibinelli-Sousa, S., Hespanhol, J. T., Nicastro, G. G., Matsuyama, B. Y., Mesnage, S., Patel, A., et al. (2020). A Family of T6SS Antibacterial Effectors Related to L,D-Transpeptidases Targets the Peptidoglycan. *Cell Rep.* 31, 107813. doi:10.1016/j.celrep.2020.107813.

Song, L., Pan, J., Yang, Y., Zhang, Z., Cui, R., Jia, S., et al. (2020). A contact-independent T6SS killing pathway mediated by a microcin-like nuclease effector possesses intrinsic cell-entry mechanisms. *Res. Sq.* doi:10.21203/rs.3.rs-65917/v1.

Tang, J. Y., Bullen, N. P., Ahmad, S., and Whitney, J. C. (2018). Diverse NADase effector families mediate interbacterial antagonism via the type VI secretion system. *J. Biol. Chem.* 293, 1504–1514. doi:10.1074/jbc.ra117.000178.

Ting, S. Y., Bosch, D. E., Mangiameli, S. M., Radey, M. C., Huang, S., Park, Y. J., et al. (2018). Bifunctional Immunity Proteins Protect Bacteria against FtsZ-Targeting ADP-Ribosylating Toxins. *Cell* 175, 1380-1392.e14. doi:10.1016/j.cell.2018.09.037.

Trunk, K., Peltier, J., Liu, Y. C., Dill, B. D., Walker, L., Gow, N. A. R., et al. (2018). The type VI secretion system deploys antifungal effectors against microbial competitors. *Nat. Microbiol.* 3, 920–931. doi:10.1038/s41564-018-0191-x.

Whitney, J. C., Beck, C. M., Goo, Y. A., Russell, A. B., Harding, B. N., De Leon, J. A., et al. (2014). Genetically distinct pathways guide effector export through the type VI secretion system. *Mol. Microbiol.* 92, 529–542. doi:10.1111/mmi.12571.

Whitney, J. C., Chou, S., Russell, A. B., Biboy, J., Gardiner, T. E., Ferrin, M. A., et al. (2013). Identification, structure, and function of a novel type VI secretion peptidoglycan glycoside hydrolase effector-immunity pair. *J. Biol. Chem.* 288, 26616–26624. doi:10.1074/jbc.M113.488320.
